# Supplementary material for: Resolution limit of image analysis algorithms
Source: Nat Commun. 2019 Feb 15;10:793. doi: 10.1038/s41467-019-08689-x (PMC6377644; doi:10.1038/s41467-019-08689-x)
Supplement: Supplementary file 1 — Supplementary Material [file 41467_2019_8689_MOESM1_ESM.pdf]

# Resolution Limit of Image Analysis Algorithms

Edward A. K. Cohen*[1], Anish V. Abraham[2,3], Sreevidhya Ramakrishnan[2,3], and Raimund J. Ober[2,3,4]

[1]Department of Mathematics, Imperial College London, London, UK

[2]Department of Biomedical Engineering, Texas A&M University, College Station, Texas, USA

[3]Department of Molecular & Cellular Medicine, Texas A&M University, College Station, TX, USA

[4]Centre for Cancer Immunology, Faculty of Medicine, University of Southampton, Southampton, UK

## Supplementary Note 1: Introduction to mathematical framework

The work presented in this paper, including the algorithmic resolution limit, the probabilistic resolution and the adjusted $K_{2\alpha}$-function, is derived through applying spatial statistical theory to imaging. In this supplementary material, we discuss this approach and formulate the mathematical framework.

We begin in Supplementary Note 2 with a review of key concepts and definitions relating to spatial point processes and patterns that will be needed to develop the mathematical framework. The paper focuses on fluorescence microscopy imaging, which we here consider to be the attempted recovery of an underlying spatial point pattern for the true locations of fluorophores. In Supplementary Note 3, we consider a mathematical formulation of the imaging process with regards to recovering the spatial point pattern. From this, we are able to reason about image resolution and define the algorithmic resolution limit, the core concept of our paper, in Supplementary Note 5. Quantifying the effect this resolution limit has on the recovery of a spatial point pattern is explored in Supplementary Note 6, where we derive the probability that an event/object (in this case, a photon-emitting fluorophore) is affected by algorithmic resolution, which we term the probabilistic resolution. In Supplementary Note 7, we apply these ideas to quantify the effect of algorithmic resolution in single molecule localization microscopy. This in turn allows us to define both a probabilistic resolution and what we term the super-resolution limit. We consider in Supplementary Note 8 a number of different scenarios in which we apply these resolution measures, one of which is a

*Correspondence should be addressed to E.A.K.C. (e.cohen@imperial.ac.uk).

tubulin data set in which we demonstrate how probabilistic resolution and the super-resolution limit can be computed.

Key to the core ideas of this paper is the ability to estimate the algorithmic resolution limit for a particular algorithm of interest from an estimate of a pair correlation function. In Supplementary Note 9, we give details for an estimator that performs this task and discuss its statistical properties.

The definition of the algorithmic resolution limit presented here does not depend on the density of the events/objects being analyzed. In Supplementary Note 10, we explore what effect event density and signal to noise ratio has on the algorithmic resolution limit. As predicted, we demonstrate with three different algorithms that the algorithmic resolution limit appears constant across a range of densities. However, a breakdown in this behavior is noticeable at high densities for some algorithms.

It will be shown that limited algorithmic resolution fundamentally changes the properties of the spatial point pattern. The altered, observed point pattern is then typically analysed with standard statistical techniques, the most common of which is Ripley's $K$-function. In Supplementary Note 11 we propose an adaptation of Ripley's $K$-function, the $K_{2\alpha}$-function, which accounts for unwanted artefacts introduced by limited algorithmic resolution. Further discussions and mathematical proofs are contained in the Appendices in Supplementary Note 12.

## Supplementary Note 2: Spatial point processes

In this section we provide some key definitions used for characterizing spatial point processes and spatial point patterns. While it is sometimes unavoidable, we refrain as much as possible from the formal measure theoretic approach. Those interested in this treatment are directed to Cressie (1993), from where much of this theory is taken.

A spatial point process is a stochastic mechanism that generates a random collection of points $\Phi = \{\mathbf{s}_1, \mathbf{s}_2, ...\}$, termed *events*, each belonging to $X$, a locally compact subset of $\mathbb{R}^d$ (typically $d = 2$ or $3$). The set of events $\Phi$ is called a spatial point pattern, and can alternatively be represented through a locally finite counting measure $\xi$ on $X$ where $\xi(B)$ is the number of events within a set $B \subseteq \mathbb{R}^d$. Formally, $B \in \mathcal{B}$ where $\mathcal{B}$ is a Borel $\sigma$-algebra on $X$.

Formally, let $(\Omega, \mathcal{F}, \mathbb{P})$ be a probability space, let $\Xi$ be a collection of locally finite counting measures on $X$ and let $\mathcal{N}$ be the smallest $\sigma$-algebra generated from sets of the form $\{\xi \in \Xi : \xi(B) = n\}$, for all $B \in \mathcal{B}$ and all $n \in \{0, 1, 2, ...\}$. A spatial point process $N$, defined on $X$, is a measurable mapping from $(\Omega, \mathcal{F})$ to $(\Xi, \mathcal{N})$. A spatial point process $N$ is therefore a random counting measure. It is typical to use notation $N(B)$ to be the random non-negative integer stating the number of events within a set $B \in \mathcal{B}$. For example, $N(\mathrm{d}\mathbf{s})$ indicates the random number of events in an infinitesimal ball $\mathrm{d}\mathbf{s}$ centred at $\mathbf{s}$. We

will restrict ourselves to orderly processes where the number of events in $\mathrm{d}s$ can only be 0 or 1.

A spatial point process can be characterized by its moment measures. The $k$th moment measure for a collection of $k$ sets $B_1, ..., B_k \in \mathcal{B}$ is

$$\mu^{(k)}(B_1 \times B_2 \times \cdots \times B_k) = E\{N(B_1)N(B_2)\cdots N(B_k)\}. \tag{1}$$

From this we can define the first and second-order intensity functions.

**Definition 1** (First-order intensity). *The first-order intensity, more commonly known as the intensity, of a spatial point process $N$ at a point $\mathrm{s} \in X$ is*

$$\lambda(\mathbf{s}) = \lim_{\nu(\mathrm{d}\mathbf{s})\downarrow 0} \frac{E\{N(\mathrm{d}\mathbf{s})\}}{\nu(\mathrm{d}\mathbf{s})} = \lim_{\nu(\mathrm{d}\mathbf{s})\downarrow 0} \frac{\mu(\mathrm{d}\mathbf{s})}{\nu(\mathrm{d}\mathbf{s})}, \tag{2}$$

*where $\nu(\cdot)$ is the Lebesgue measure.*

We interpret the first-order intensity as being the expected density of events per unit volume generated by the process at a particular $\mathrm{s} \in X$. Equivalently, we have the relationship $\lambda(\mathbf{s})\nu(\mathrm{d}\mathbf{s}) = \mathbb{P}(N(\mathrm{d}\mathbf{s}) = 1)$.

**Definition 2** (Second-order intensity). *The second-order intensity of a spatial point process $N$ at points $\mathrm{s}, \mathbf{u} \in X$ is*

$$\gamma(\mathbf{s}, \mathbf{u}) = \lim_{\nu(\mathrm{d}\mathbf{s})\nu(\mathrm{d}\mathbf{u})\downarrow 0} \frac{E\{N(\mathrm{d}\mathbf{s})N(\mathrm{d}\mathbf{u})\}}{\nu(\mathrm{d}\mathbf{s})\nu(\mathrm{d}\mathbf{u})} = \lim_{\nu(\mathrm{d}\mathbf{s})\nu(\mathrm{d}\mathbf{u})\downarrow 0} \frac{\mu^{(2)}(\mathrm{d}\mathbf{s} \times \mathrm{d}\mathbf{u})}{\nu(\mathrm{d}\mathbf{s})\nu(\mathrm{d}\mathbf{u})} \tag{3}$$

The second-order intensity provides a route into looking at correlations in the process across a distance. For such purposes, the auto-covariance is given as $c(\mathbf{s}, \mathbf{u}) = \gamma(\mathbf{s}, \mathbf{u}) - \lambda(\mathbf{s})\lambda(\mathbf{u})$ and the auto-correlation $c(\mathbf{s}, \mathbf{u})/\lambda(\mathbf{s})\lambda(\mathbf{u})$. A related measure to characterize second-order interactions in a point process is the pair correlation function.

**Definition 3** (Pair correlation function). *The pair correlation function of a spatial point process $N$ at points $\mathrm{s}, \mathbf{u} \in X$ is*

$$g(\mathbf{s}, \mathbf{u}) = \frac{\gamma(\mathbf{s}, \mathbf{u})}{\lambda(\mathbf{s})\lambda(\mathbf{u})}. \tag{4}$$

Two important notions that arise from these definitions are that of stationarity and isotropy.

**Definition 4** (Second order stationarity and Isotropy). *A point process is second-order stationary (from here-on referred to as stationary) if $\gamma(\mathbf{s}, \mathbf{u}) = \gamma(\mathbf{s} - \mathbf{u})$ and isotropic if $\gamma(\mathbf{s}, \mathbf{u}) = \gamma(\|\mathbf{s} - \mathbf{u}\|)$.*

For a stationary and isotropic process, $\gamma(\cdot)$ and $g(\cdot)$ simply become functions of $r \in \mathbb{R}$, the Euclidean distance between two points.

It is essential to define the Poisson process. Many definitions exist, we choose the following for its simplicity.

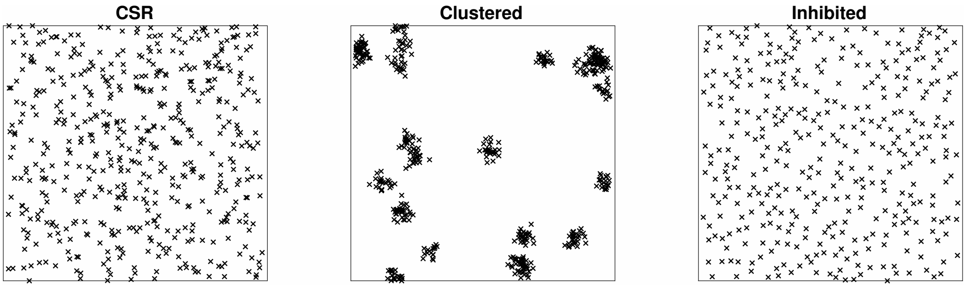


**Supplementary Figure 1.** Example realizations or a completely spatially random, clustered and inhibited point pattern.

**Definition 5** (Poisson Process)**.** *Spatial point process $N$, defined on $X \subseteq \mathbb{R}^d$, is Poisson if the following hold:*

1. *For every $B \in \mathcal{B}$, the number of events is Poisson distributed with expected value $\mu(B) = \int_B \lambda(\mathbf{s})\mathrm{d}\nu(\mathbf{s})$, i.e.*
$$\mathbb{P}(N(B) = n) = \frac{\mu(B)^n}{n!}\exp(-\mu(B)). \tag{5}$$
2. *For any collection $B_1, ..., B_n$ of disjoint sets from $\mathcal{B}$, $N(B_1), ..., N(B_n)$ are independent random variables.*

Poisson processes for which $\lambda(\mathbf{s})$ is constant on $X$ are called homogeneous and are both stationary and isotropic. Poisson processes possess the important memoryless property. That is to say, all events are independent of each other and there is no correlation across space. For this reason, homogeneous Poisson processes (HPP) are commonly termed *completely spatially random* (CSR). In general, the deviations from complete spatial randomness come in two forms. In clustered patterns, events lie closer to each other than one would expect for CSR data. For inhibited patterns, events lie further away from each other than one would expect for CSR data. Example realizations of CSR, clustered and inhibited patterns are shown in Supplementary Figure 1. These ideas can be rigorously formulated by reasoning about spatial correlation structure within the data.

Ripley's $K$-function is used extensively across the sciences, including microscopy (Lagache et al., 2013; Rossy et al., 2014), as a method for analyzing spatial point patterns, primarily to detect and characterize clustering behavior. Its widespread use lies in its interpretability and the ease at which it can be estimated with robust, well studied estimators.

**Definition 6** (Ripley's $K$-function)**.** *For a stationary and isotropic process, with pair correlation function*

$g(\mathbf{s}, \mathbf{u}) = g(r)$, *Ripley's $K$-function is defined as*

$$K(r) \equiv \int_0^r 2\pi r' g(r') \mathrm{d}r'$$
$$= \lambda^{-1} E\{\textit{number of events within distance } r \textit{ of an arbitrary event}\}. \tag{6}$$

The pair correlation function and Ripley's $K$-function are used to characterize the notion of spatial randomness, clustering and regularity. Complete spatial randomness has the property that $g(r) = 1$ for all $r > 0$, and consequently $K(r) = \pi r^2$. If $g(r) > 1$ for some $r$ then that indicates we are more likely to see an event at a distance $r$ from an arbitrary event than under complete spatial randomness. If $g(r') > 1$ for all $r' \in (0, r]$ then $K(r) > \pi r^2$. Such second-order behavior gives rise to clusters. Alternatively, if $g(r) < 1$ then events are less likely to be found at a distance $r$ from one another than you would expect under complete spatial randomness, and if $g(r') < 1$ for all $r' \in (0, r]$ then $K(r) < \pi r^2$. It is processes of this type that are called inhibited (or regular).

It is common to define $L(r) = \sqrt{K(r)/\pi}$ which linearises the behavior of the $K$-function such that $L(r) = r$ for an HPP, is greater than $r$ for a clustered process and less than $r$ for a regular process. Further to this, it is common to compute and analyze the $L(r) - r$ function which is equal to zero for an HPP, greater than zero for a clustered process, and less than zero for a regular process.

We further introduce another important summary property of a point process, the nearest neighbor distribution function.

**Definition 7** (Nearest neighbor distribution function)**.** *For a stationary process, let $D$ denote the distance from an arbitrary event to the nearest other event. The nearest neighbor distribution (NND) function is defined as*

$$G(r) \equiv \mathbb{P}(D \leq r). \tag{7}$$

Letting $b(\mathbf{s}, r) \subset X$ denote a ball of radius $r$ centred at $\mathbf{s}$, an equivalent expression for the NDD function is $G(r) = 1 - \mathbb{P}(N(b(\mathbf{s}, r)) = 1 | N(\mathrm{d}\mathbf{s}) = 1)$, where $\mathbb{P}(N(b(\mathbf{s}, r)) = 1 | N(\mathrm{d}\mathbf{s}) = 1)$ can be read as the probability that apart from the event at $\mathbf{s}$ there are no further events in $b(\mathbf{s}, r)$. For a stationary process, this does not depend on the location $\mathbf{s}$. For an HPP we have $G(r) = 1 - \exp(-\lambda \pi r^2)$.

In what follows, it will become necessary to consider Cox processes, sometimes referred to as doubly stochastic processes.

**Definition 8** (Cox process)**.** *Point process $N$ is a Cox process driven by a random intensity function $\Lambda(\mathbf{s})$ if, conditioning on $\Lambda(\mathbf{s}) = \lambda(\mathbf{s})$, $N$ is an inhomogeneous Poisson process with intensity $\lambda(\mathbf{s})$.*

To understand the Cox process, it can be useful to consider that a realization firstly involves a realization

$\lambda(\mathbf{s})$ of the random intensity function $\Lambda(\mathbf{s})$, and then a realization of a Poisson process with intensity $\lambda(\mathbf{s})$. The first and second-order properties of a Cox process are intrinsically linked to the first and second order properties of the random intensity $\Lambda(\mathbf{s})$, with a Cox process $N$ being second-order stationary (as defined in Definition 4) if and only if $E\{\Lambda(\mathbf{s})\}$ is constant for all $\mathbf{s} \in X$ and $E\{\Lambda(\mathbf{s})\Lambda(\mathbf{u})\}$ depends only on $\mathbf{s} - \mathbf{u}$. Cox processes represent a broad class of spatial point processes, including certain types of cluster process and fibre driven Cox processes (Illian et al., 2008, p. 383); point processes where events a generated on randomly placed fibres (curves). These ideas and their use in this setting will be discussed further in Supplementary Note 8.

## Supplementary Note 3: Imaging point processes

We consider imaging a collection of objects whose positions we model as a spatial point pattern. The key philosophy of the presented work is treating the imaging procedure as the attempted recovery of this spatial point pattern and we judge an imaging system by how well it performs this recovery. In this section we consider what effect the imaging procedure, which in its most general form includes an optical system and a sequence of one or more image processing algorithms, has on the point pattern we are attempting to image. The fundamental property which we wish to consider is that of resolution - i.e. how well does the imaging procedure deal with objects (events) that are close to one another. In doing so we begin with defining the object point process, the imaging operator and the image point process. We refer the reader to Supplementary Figure 2 which illustrates this definition.

**Definition 9** (Object point process/pattern, imaging operator and image point process/pattern)**.** *The object point process $N_{\mathrm{O}}$ that maps a probability space $(\Omega, \mathcal{F})$ to $(\Xi_{\mathrm{O}}, \mathcal{N}_{\mathrm{O}})$ is the unobserved spatial point process for the objects being imaged. The associated unobserved object point pattern is represented by event set $\Phi_{\mathrm{O}}$. The imaging operator $I$ is a mapping from $(\Xi_{\mathrm{O}}, \mathcal{N}_{\mathrm{O}})$ to some space $(\Xi_I, \mathcal{N}_I)$. The image spatial point process $N_I$ is the combined mapping $I \circ N_{\mathrm{O}}$ from $(\Omega, \mathcal{F})$ to $(\Xi_I, \mathcal{N}_I)$. The observed image point pattern is represented by event set $\Phi_I$.*

We will call $I$ a *stationary and isoptropic* imaging operator if it preserves stationarity and isotropy, i.e. $N_I$ is stationary and isotropic if $N_{\mathrm{O}}$ is stationary and isotropic. For the purposes of this paper, we will assume all imaging operators are stationary and isotropic.

We propose that resolution is a property of an imaging operator and consider two possible criteria that can be used as a metric for it. We begin by examining an idealized imaging operator in which there exists a hard detection resolution limit such that within it two events are not individually resolved, and outside they are resolved. We then consider a more applicable resolution limit, termed the algorithmic resolution limit.

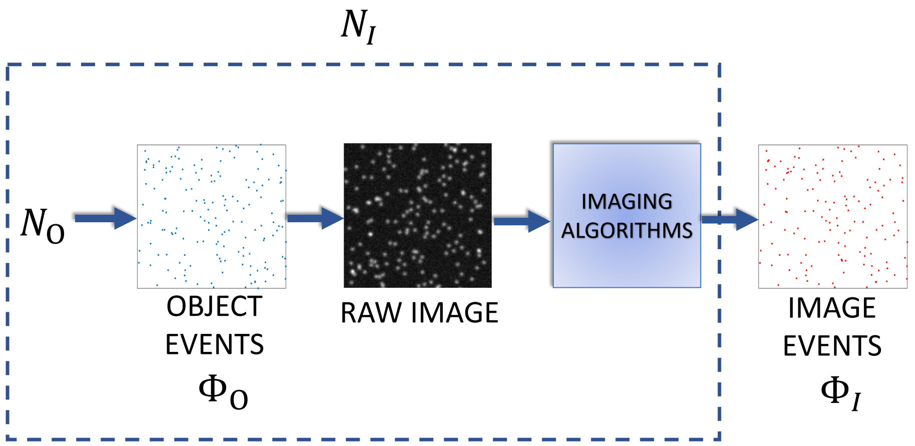


**Supplementary Figure 2.** Schematic illustrating the stages in realizing a image pattern. Point process $N_{\mathrm{O}}$ gives rise to a point pattern $\Phi_{\mathrm{O}}$. These light emitting molecules produce a raw image which is processed through a series of segmentation and localization algorithms. The collection of locations output by the system is the image point pattern $\Phi_I$. The collection of steps that give rise to the image pattern can be considered to be the image process $N_I$.

## Supplementary Note 4: Detection resolution limit

We consider the following idealized imaging operator which we denote $I_\delta$.

1. If two events from $N_{\mathrm{O}}$ are within a distance $\delta > 0$ of each other we observe just one of the events.
2. The event we observe is that which is most "prominent". (Prominence could, for example, be the intensity of the imaged event).

A mathematical formulation of this point process is as follows.

**Model 1** (Imaging operator $I_\delta$)**.** *Let $N_{\mathrm{O}}$ be a point process,*

1. *each event $\mathbf{s} \in \Phi_{\mathrm{O}}$ generated by the process $N_{\mathrm{O}}$ is also assigned a random mark $Z(\mathbf{s})$ with distribution function $F(\cdot)$ (which acts as its prominence/intensity),*
2. *an event $\mathbf{s}$ is removed if there exists another event $\mathbf{u}$ from $N_{\mathrm{O}}$ with $||\mathbf{s}-\mathbf{u}|| < \delta$ and $Z(\mathbf{s}) < Z(\mathbf{u})$.*

This process was originally formulated in Matérn (1960) for the case of $N_{\mathrm{O}}$ being an HPP and marks $Z \sim$ Uniform$(0,1)$, and is known as the Matérn II process. It was shown in Stoyan and Stoyan (1985) that the process is invariant to the choice of distribution function $F$, so generalizes to this application.

When $N_{\mathrm{O}}$ is an HPP with intensity $\lambda_{\mathrm{O}}$, the intensity of the remaining process $N_{I_\delta}$ is given in Diggle (1983) as

$$\lambda_{I_\delta} = \frac{1-\exp(-\lambda_{\mathrm{O}}\pi\delta^2)}{\pi\delta^2}, \tag{8}$$

and the second-order intensity of $N_{I_\delta}$ is

$$\gamma_{I_\delta}(r) = \begin{cases} 0 & r < \delta \\ \frac{2U_\delta(r)\{1-\exp(\lambda_{\mathrm{O}}\pi\delta^2)\}-2\pi\delta^2\{1-\exp(-\lambda_{\mathrm{O}}U_\delta(r))\}}{\pi\delta^2 U_\delta(r)\{U_\delta(r)-\pi\delta^2\}} & \delta \leq r < 2\delta \\ \lambda_{I_\delta}^2 & r \geq 2\delta, \end{cases} \quad (9)$$

where $U_\delta(r) = 2\pi\delta^2 - 2\delta^2 \arccos(r/(2\delta)) + \frac{1}{2}r(4\delta^2 - r^2)^{1/2}$ is the area of the union of two circles of diameter $\delta$ a distance $r$ apart. This states that when $N_{\mathrm{O}}$ is an HPP, and hence $N_{\mathrm{O}}(\mathrm{d}\mathbf{s})$ is uncorrelated with $N_{\mathrm{O}}(\mathrm{d}\mathbf{u})$ for all $\mathbf{s} \neq \mathbf{u}$, the imaging operator introduces correlation into the image process $N_{I_\delta}$ that occurs over a distance of $2\delta$. Examples of the theoretical Ripley's $K$-function and pair correlation function for models of this type can be found in Supplementary Figure 3B.

For a general $N_{\mathrm{O}}$ imaged with this idealized imaging operator $I_\delta$, the second-order intensity $\gamma_\delta$ is intractable. However, we can say with certainty that

$$\gamma_{I_\delta}(r) \begin{cases} = 0 & r < \delta \\ > 0 & r \geq \delta, \end{cases} \quad (10)$$

from which we can also say the pair correlation function $g_{I_\delta}(r)$, Ripley's $K$-function $K_{I_\delta}(r)$ and NND function $G_{I_\delta}(r)$ have the form

$$g_{I_\delta}(r) \begin{cases} = 0 & r < \delta \\ > 0 & r \geq \delta, \end{cases} \qquad K_{I_\delta}(r) \begin{cases} = 0 & r < \delta \\ > 0 & r \geq \delta, \end{cases} \qquad G_{I_\delta}(r) \begin{cases} = 0 & r < \delta \\ > 0 & r \geq \delta. \end{cases} \quad (11)$$

In this idealized case, we call $\delta$ the detection resolution limit (DRL) and can mathematically formulate it with the following definition.

**Definition 10.** *The detection resolution limit (DRL) $\delta$ of an imaging system is given as*

$$\begin{aligned} \delta &= \sup\{r : g_{I_\delta}(r) = 0\} \\ &= \sup\{r : K_{I_\delta}(r) = 0\} \\ &= \sup\{r : G_{I_\delta}(r) = 0\}. \end{aligned} \quad (12)$$

No two events in the pattern $\Phi_{I_\delta}$, generated by process $N_{I_\delta}$, can be within a distance $\delta$ of each other. Supplementary Figure 3 gives further details on this model, including theoretical pair correlation and Ripley's $K$-functions under different $\delta$.

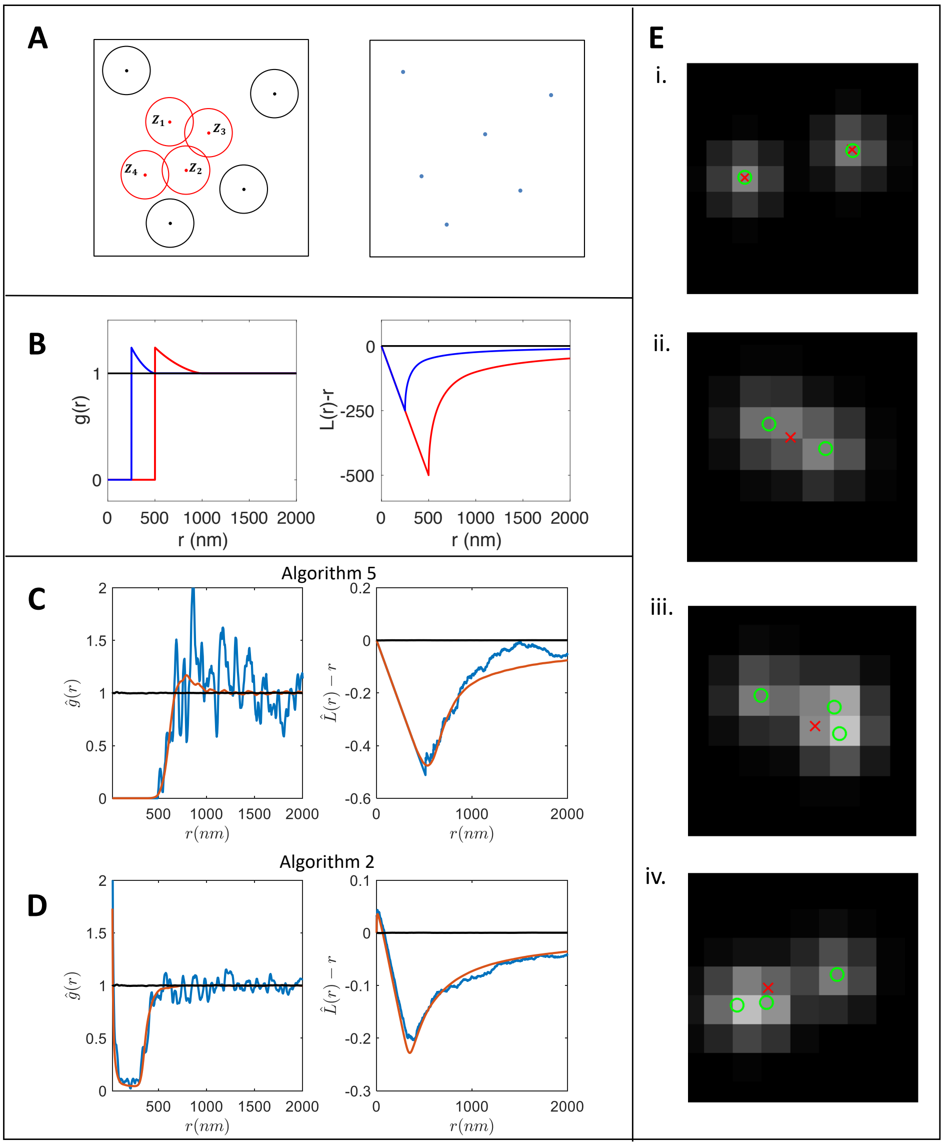


**Supplementary Figure 3.** **A**. Illustration of the effect of resolution under a classical detection resolution model for the imaging operator – see Supplementary Note 4. Left: each point represents an event of the object pattern $\Phi_O$ with a circle of radius $\delta/2$ round each one. Events whose circles intersect with another circle (shown in red) will be affected by resolution. We associate with each event a random mark $Z_i$ (we label only the marks for the events in red). The red events form a resolution limited sub-pattern (RSLP) of size 4 – see Defintion 13. Right: the resulting image point pattern $\Phi_{I_\delta}$ in the case where $Z_1 < Z_2 < Z_3 < Z_4$. Irrespective of the values of the marks, events in $\Phi_I$ will always be further than $\delta$ away from any other event. **B**. Left: the theoretical pair correlation function and right: the theoretical $L(r) - r$ function for idealized detection resolution limited HPP subject to detection resolution limits of 250nm (blue) and 500nm (red). **C**. Single estimate (blue) and averaged estimate (red) of Left: pair correlation function and Right: $L(r) - r$ function for an HPP localized with Algorithm 5. See *Methods* for simulation and analysis details. **D**. Single estimate (blue) and averaged estimate (red) of Left: pair correlation function and Right: $L(r) - r$ function for an HPP localized with Algorithm 2. The black curve represents the averaged ground truth. See the *Methods* for simulation and analysis details. **E**. Four segments of simulated images. True locations of emitters (events in $\Phi_O$) are marked with green circles and estimated locations of emitters (events in $\Phi_I$) are marked with red crosses. i. Two emitters well separated in space and not affected by resolution. ii. Two emitters in close enough proximity to each other as to be affected by resolution. iii. - iv. Three emitters, each close enough to one of the other emitters as to be affected by resolution. See *Methods* for details of simulations. Algorithm 1 was used as the analysis method.

## Supplementary Note 5: Algorithmic resolution

In an experimental setting, although we sometimes see the clear presence of a DRL (for example in the case of Algorithm 5, see Supplementary Figure 3C), it is also common to see behavior that is uncharacteristic of this modelling assumption and where a DRL is not apparent (for example when using Algorithm 2, see Supplementary Figure 3D). In general, therefore, the DRL is an inappropriate measure of resolution and we seek to give a resolution limit that has physical meaning, can be readily estimated from data and effectively characterizes the performance of the image processing algorithms used, i.e. the imaging operator. We do this through defining the algorithmic resolution limit.

We motivate the algorithmic resolution limit by considering an idealized but more general imaging operator than that considered in Supplementary Note 4. Let $N_{\mathrm{O}}$ be a general object process generating object point pattern $\Phi_{\mathrm{O}}$. We consider the following rules for the stationary and isotropic imaging operator $I_\alpha$, resulting in image process $N_{I_\alpha} = I_\alpha \circ N_{\mathrm{O}}$ that generates pattern $\Phi_{I_\alpha}$.

**Model 2** (Imaging operator $I_\alpha$)**.** *Let $N_{\mathrm{O}}$ be a point process,*

1. *if an event $\mathbf{s} \in \Phi_{\mathrm{O}}$ generated by $N_{\mathrm{O}}$ has no other event within a distance $\alpha$ of it then $\mathbf{s} \in \Phi_{I_\alpha}$ with probability 1,*
2. *if an event $\mathbf{s} \in \Phi_{\mathrm{O}}$ generated by $N_{\mathrm{O}}$ has one or more events within a distance $\alpha$ of it then $\mathbf{s} \in \Phi_{I_\alpha}$ with probability $p < 1$.*

Here, $I_\alpha$ is a more general class of imaging operator than $I_\delta$. Indeed, the class of operators of type $I_\delta$ is a subclass of operators of type $I_\alpha$, where $\alpha = \delta$. We saw in Supplementary Note 4 that when applied to an HPP (i.e. a completely spatially random process that has no correlation across distance), $N_{\mathrm{O}}$ introduces correlation into the image process $N_{I_\delta}$ that ranges over a distance of $2\delta$. Building on this, we now have the following result for operators of type $I_\alpha$ as defined in Model 2.

**Proposition 1.** *Let $N_{\mathrm{O}}$ be an HPP and let $I_\alpha$ be the stationary and isotropic imaging operator stated in Model 2. Resulting image process $N_{I_\alpha} = I \circ N_{\mathrm{O}}$ exhibits correlation over a distance of $2\alpha$, i.e. $N_{I_\alpha}(\mathrm{d}\mathbf{s})$ is uncorrelated with $N_{I_\alpha}(\mathrm{d}\mathbf{u})$ if and only if $\|\mathbf{s} - \mathbf{u}\| \geq 2\alpha$.*

The proof to this proposition is given in Supplementary Note 12.3. In both Model 1 and Model 2, the correlation introduced into $N_I$ by the respective imaging operator acts over twice the distance at which resolution has an effect ($\delta$ and $\alpha$, respectively). This correlation manifests itself in the pair correlation function. For an HPP $N_{\mathrm{O}}$, the pair correlation function is a constant 1 for all $r > 0$ (indicating zero correlation for all $r > 0$), however, for $N_I$ it will have a deviation from this constant 1 over the interval $(0, 2\alpha)$ (indicating positive or negative correlation). While $I_\alpha$ is a specific model for the imaging operator

and algorithms used in real life are unlikely to conform exactly to this model, we use this as justification for our definition of the imaging operator. A more realistic, yet analytically intractable model for the imaging operator is given consideration in Supplementary Note 12.1. It provides further justification for the definition of the algorithmic resolution limit we now present.

To formally define the algorithmic resolution limit we introduce the radius of correlation of an imaging operator.

**Definition 11.** *Let $\Phi_{\mathrm{O}}$ be a point pattern generated by HPP $N_{\mathrm{O}}$, and let $I$ be a stationary and isotropic imaging operator that acts on $N_{\mathrm{O}}$ to produce $N_I$ with associated pattern $\Phi_I$. The radius of correlation $\rho$ of $I$ is given as:*

$$\rho := \inf_{r>0}\{r : N_I(\mathrm{d}\mathbf{s}) \text{ is uncorrelated with } N_I(\mathrm{d}\mathbf{u}) \text{ for all } \|\mathbf{s}-\mathbf{u}\| > r\}. \tag{13}$$

*An equivalent definition is given as*

$$\rho := \inf_{r>0}\{r : g_I(r') = 1 \text{ for all } r' \in [r, \infty)\}. \tag{14}$$

This distance is sometimes referred to as the *range of correlation* of $N_I$ (Illian et al., 2008), and given $N_I$ is the composition mapping of $I$ on $N_{\mathrm{O}}$, where $N_{\mathrm{O}}$ is completely spatially random, we can interpret it as being the range of correlation introduced by $I$ into $N_I$. We now formally define the algorithmic resolution limit (ARL).

**Definition 12.** *Let $I$ be a stationary and isotropic imaging operator with radius of correlation $\rho$. The algorithmic resolution limit (ARL) $\alpha$ of $I$ is*

$$\alpha := \frac{\rho}{2}. \tag{15}$$

Through defining the ARL of an imaging operator $I$ as half its radius of correlation, we are able to generalize the resolution limit to imaging operators that display a wide range of characteristics and artefacts.

We briefly note that all localizations are subject to measurement error, however the ARL naturally accounts for this. It is shown in Supplementary Note 12.2 that the general spatial structure of point processes are unaffected by localization errors. In particular, a CSR process remains CSR under localization error. Therefore, if an imaging operator perfectly resolves the HPP but with independent and identically distributed (iid) errors on all localizations, its ARL is still 0. An estimation procedure for the ARL is presented in Supplementary Note 9.

## Supplementary Note 6: Quantifying the effect of resolution

We look to quantify the effect $I$ has on $N_{\mathrm{O}}$ in producing the image process $N_I$. To do so it becomes necessary to define a *resolution limited sub-pattern* (RLSP) - an illustration of which is given in Supplementary Figure 4.

**Definition 13** (Resolution limited sub-pattern). *Let the* $\Phi_{\mathrm{O}} = \{\mathbf{s}_1, \mathbf{s}_2, ..., \}$ *be a point pattern generated by process* $N_{\mathrm{O}}$ *and let* $\alpha$ *be the ARL of a stationary and isotropic imaging operator* $I$. *A resolution limited sub-pattern* $\mathcal{S} \subseteq \Phi_{\mathrm{O}}$, $|\mathcal{S}| \geq 2$, *is a subset of the object pattern* $\Phi_{\mathrm{O}}$ *such that all its members are within distance* $\alpha$ *of at least one other member, not including itself. That is to say, for all* $\mathbf{s}_i \in \mathcal{S}$ *there exists* $\mathbf{s}_j \in \mathcal{S}$, $\mathbf{s}_i \neq \mathbf{s}_j$, *such that* $\|\mathbf{s}_i - \mathbf{s}_j\| < \alpha$.

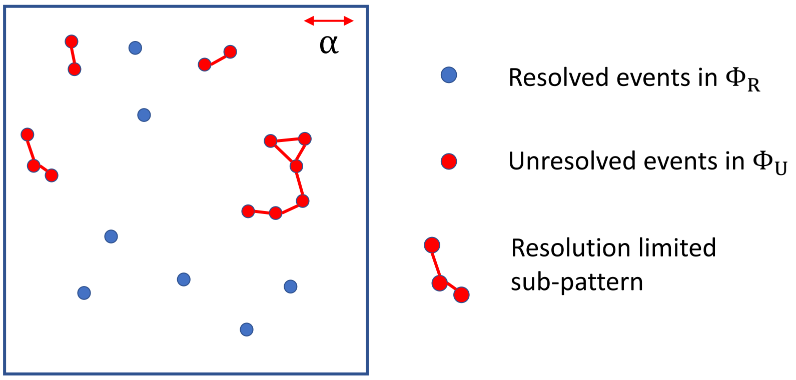


**Supplementary Figure 4.** Illustration of how a resolution limit $\alpha$ gives rise to resolved events, unresolved events and resolution limited sub-patterns.

An event that belongs to an RLSP is affected by resolution due to its proximity to another event in that same RLSP. All events that do not belong to an RLSP are not affected by resolution and are resolved by imaging operator $I$. For a given $\alpha$, the number of RSLPs, $M(\alpha)$, is a random variable that is realized with each realization of the point pattern. For a pattern $\Phi_{\mathrm{O}}$ and $\alpha > 0$, the corresponding RSLPs $\mathcal{S}_1, ..., \mathcal{S}_{M(\alpha)}$ will, by construction, have properties $\mathcal{S}_i \cap \mathcal{S}_j = \varnothing$ for all $i, j = 1, ..., M(\alpha)$, $i \neq j$, and $\bigcup_{i=1}^{M(\alpha)} \mathcal{S}_i \subseteq \Phi_{\mathrm{O}}$.

As an aside; we can draw analogies here with *random geometric graphs* (Penrose, 2003). Random geometric graphs are graphical models where nodes are randomly distributed in space and an edge is formed between two nodes if and only if the distance between those two nodes is less than a pre-defined *neighborhood distance*. Under this formulation, we can consider an event of the pattern to be a node and the resolution limit $\alpha$ to be the neighborhood distance. Two events are unresolvable if they are within distance $\alpha$ of each other, i.e. if an edge exists between them. An RLSP, in graphical models terminology, would therefore be a component of order greater than one. The work of Penrose (2003) looks

at distributions for the number of components, however, the results are asymptotic and highly technical and we elect to leave out such analysis.

### 6.1 The resolved and unresolved processes

Events which are further than a distance $\alpha$ from any other event can be completely resolved and belong to the *resolved point pattern* $\Phi_{\mathrm{R}} \subseteq \Phi_{\mathrm{O}}$. We say the mechanism generating this is the *resolved point process* $N_{\mathrm{R}}$. The resolved events are all those that do not belong to an RSLP, and therefore $\Phi_{\mathrm{R}} = \Phi_{\mathrm{O}} \setminus \bigcup_{i=1}^{M(\alpha)} \mathcal{S}_i$. Events that are within a distance $\alpha$ of another event are affected by resolution and form the *unresolved point pattern* $\Phi_{\mathrm{U}}$ generated by *unresolved point process* $N_{\mathrm{U}}$. Pattern $\Phi_{\mathrm{U}}$ is comprised of all the events in $\Phi_{\mathrm{O}}$ that belong to an RSLP, i.e. $\Phi_{\mathrm{U}} = \bigcup_{i=1}^{M(\alpha)} \mathcal{S}_i$. It is clear that we have $N_{\mathrm{O}} = N_{\mathrm{R}} + N_{\mathrm{U}}$ and $\Phi_{\mathrm{O}} = \Phi_{\mathrm{R}} \cup \Phi_{\mathrm{U}}$.

For a stationary process $N_{\mathrm{O}}$ with intensity function $\lambda_{\mathrm{O}}$, the intensity $\lambda_{\mathrm{R}}$ of the resolved process $N_{\mathrm{R}}$ is given as

$$\begin{aligned}
\lambda_{\mathrm{R}}(\mathbf{s})\nu(\mathrm{d}\mathbf{s}) &= \mathbb{P}(N_{\mathrm{R}}(\mathrm{d}\mathbf{s}) = 1) \\
&= \mathbb{P}(N_{\mathrm{R}}(\mathrm{d}\mathbf{s}) = 1 | N_{\mathrm{O}}(\mathrm{d}\mathbf{s}) = 1)\mathbb{P}(N_{\mathrm{O}}(\mathrm{d}\mathbf{s}) = 1) \\
&= \mathbb{P}(N_{\mathrm{O}}(b(\mathbf{s},\alpha)) = 1 | N_{\mathrm{O}}(\mathrm{d}\mathbf{s}) = 1)\lambda_{\mathrm{O}}\nu(\mathrm{d}\mathbf{s}). \\
&= (1 - G_{\mathrm{O}}(\alpha))\lambda_{\mathrm{O}}\nu(\mathrm{d}\mathbf{s}). \qquad (16)
\end{aligned}$$

Recognising that $\lambda_{\mathrm{O}} = \lambda_{\mathrm{R}} + \lambda_{\mathrm{U}}$, where $\lambda_{\mathrm{U}}$ is the intensity of the unresolved process $N_{\mathrm{U}}$,

$$\lambda_{\mathrm{R}} = \lambda_{\mathrm{O}}(1 - G_{\mathrm{O}}(\alpha)) \qquad (17)$$

$$\lambda_{\mathrm{U}} = \lambda_{\mathrm{O}} G_{\mathrm{O}}(\alpha). \qquad (18)$$

Supplementary Figure 4 illustrates the notion of RLSPs and resolved and unresolved processes.

### 6.2 Homogeneous Poisson object process

In the situation where the object process $N_{\mathrm{O}}$ is an HPP, the resolved process $N_{\mathrm{R}}$ takes the form of a well studied model of point process. This model was first proposed by Matérn (1960) and given formal treatment in Cressie (1993); Diggle (1983). It is known commonly as Matérn's Model I, or Matérn's hardcore process I. For an HPP object process $N_{\mathrm{O}}$ with intensity $\lambda_{\mathrm{O}}$, if we exclude any events that have a neighbor within some predefined *hardcore* distance, then the remaining process is Matérn I. This is exactly what we have in the case of the resolved process $N_{\mathrm{R}}$, where $\alpha$ is the hardcore distance. With the NDD function of $N_{\mathrm{O}}$ given as $G(r) = 1 - \exp(-\lambda_{\mathrm{O}}\pi r^2)$, from (17) and (18), the intensities $\lambda_{\mathrm{R}}$ and $\lambda_{\mathrm{U}}$ of $N_{\mathrm{R}}$

and $N_{\mathrm{U}}$, respectively, become

$$\lambda_{\mathrm{R}} = \lambda_{\mathrm{O}} \exp(-\lambda_{\mathrm{O}} \pi \alpha^2) \tag{19}$$

$$\lambda_{\mathrm{U}} = \lambda_{\mathrm{O}} (1 - \exp(-\lambda_{\mathrm{O}} \pi \alpha^2)). \tag{20}$$

The second-order intensity function of $N_{\mathrm{R}}$ is given in Diggle (1983) as

$$\gamma_{\mathrm{R}}(r) = \begin{cases} 0 & r < \alpha \\ \lambda_{\mathrm{R}}^2 \exp(\lambda_{\mathrm{O}} R_\alpha(r)) & \alpha \leq r \leq 2\alpha \\ \lambda_{\mathrm{R}}^2 & r > 2\alpha, \end{cases} = \begin{cases} 0 & r < \alpha \\ \lambda_{\mathrm{O}}^2 \exp(-\lambda_{\mathrm{O}} U_\alpha(r)) & \alpha \leq r \leq 2\alpha \\ \lambda_{\mathrm{R}}^2 & r > 2\alpha, \end{cases} \tag{21}$$

where $R_\alpha(r) = 2\alpha^2 \arccos(r/(2\alpha)) - \frac{1}{2} r (4\alpha^2 - r^2)^{1/2}$ is the area of the intersection and $U_\alpha(r) = 2\pi\alpha^2 - R_\alpha(r)$ is the area of the union of two circles of diameter $\alpha$ with centers a distance $r$ apart.

### 6.3 Probabilistic resolution

We can now make statements of probability regarding whether an event from the object process $N_{\mathrm{O}}$ is affected by resolution or not. Reasoning about resolution in terms of probabilities is a useful exercise because the effect of resolution on a point pattern $\Phi_{\mathrm{O}}$ is fundamentally dependent on the nature of the point process $N_{\mathrm{O}}$. To motivate this idea, consider an inhibited point process. For example, suppose that $N_{\mathrm{O}}$ is itself a Matérn I process with hardcore distance greater than $\alpha$. All events from $N_{\mathrm{O}}$ will be further than $\alpha$ away from each other and the entire point pattern will be perfectly resolved. In contrast, consider a heavily clustered process where each event has a high probability of having another event within distance $\alpha$ of it, then a large proportion of events will be affected by resolution and the point pattern will be poorly resolved. It is this difference in the resolvability of a point pattern that we wish to capture with probabilistic resolution.

To formalize this, we say an event $\mathbf{s} \in \Phi_{\mathrm{O}}$ is affected by resolution if, for a resolution limit $\alpha$, $\mathbf{s} \in \Phi_{\mathrm{U}}$. Conversely, we say it is unaffected by resolution if $\mathbf{s} \in \Phi_{\mathrm{R}}$. We capture this with the probabilistic resolution.

**Definition 14.** *Given a resolution limit $\alpha$, the probabilistic resolution of a point process $N_{\mathrm{O}}$ is the probability of an event being unaffected by resolution,*

$$p_{N_{\mathrm{O}},\alpha} \equiv \mathbb{P}(\mathbf{s} \in \Phi_{\mathrm{R}} | \mathbf{s} \in \Phi_{\mathrm{O}}). \tag{22}$$

**Proposition 2.** *The probabilistic resolution of a point process $N_{\mathrm{O}}$ imaged under resolution limit $\alpha$ is*

$$p_{N_{\mathrm{O}},\alpha} = 1 - G_{\mathrm{O}}(\alpha). \tag{23}$$

The result of Proposition 2 is obtained directly from Supplementary Equation (17). Clearly, the probability of an event being affected by resolution is $G_{\mathrm{O}}(\alpha)$.

## Supplementary Note 7: Quantifying the effect of resolution in single molecule localization microscopy

We now demonstrate how probabilistic resolution can be used to provide a measure of resolution in single molecule localization microscopy (SMLM). In an SMLM experiment only a sparse subset of the events (molecules) from the object process $N_{\mathrm{O}}$ are in the photon-emitting (On) state in any given frame, and therefore the probability an event is affected by resolution (i.e. is within a distance $\alpha$ of another molecule in the On state) is lower than it would be if all the molecules were on at the same time. We can use the concept of resolution limited point patterns to rigorously quantify this probability and use Definition 14 to provide a measure of resolution.

### Idealized SMLM experiment

Consider an SMLM experiment in which the super-resolved image is formed through the capture of $n_{\mathrm{F}}$ successive frames. We represent this as a random partition $\{\Phi^1, ..., \Phi^{n_{\mathrm{F}}}\}$ of $\Phi_{\mathrm{O}}$, with the modelling assumptions:

1. every molecule appears in exactly one frame,

$$\bigcup_{i=1}^{n_{\mathrm{F}}} \Phi^i = \Phi_{\mathrm{O}},$$
$$\Phi^i \cap \Phi^j = \varnothing, \text{ for all } i, j = 1, ..., n_{\mathrm{F}},\ i \neq j, \tag{24}$$

2. the molecules are evenly divided amongst frames

$$\mathbb{P}(\mathbf{s} \in \Phi^i | \mathbf{s} \in \Phi_{\mathrm{O}}) = n_{\mathrm{F}}^{-1} \quad \text{for all } i = 1, ..., n_{\mathrm{F}}, \tag{25}$$

3. molecules are independently divided among frames

$$\mathbb{P}(\mathbf{s} \in \Phi^i | \mathbf{u} \in \Phi^j) = \mathbb{P}(\mathbf{s} \in \Phi^i) \quad \text{for all } \mathbf{s}, \mathbf{u} \in \Phi_{\mathrm{O}},\ i, j = 1, ..., n_{\mathrm{F}}. \tag{26}$$

We say the spatial point pattern in each frame is $q$-thinned where $q = n_{\mathrm{F}}^{-1}$, i.e. the probability that an event (molecule) appears in a specific frame is $q$. For each frame pattern $\Phi^i$, under stationary and isotropic imaging operator $I$ with ARL $\alpha$, we observe the image point pattern $\Phi^i_I$. Associated with this is the

corresponding resolved pattern $\Phi_{\mathrm{R}}^i$ and the unresolved pattern $\Phi_{\mathrm{U}}^i$. The collection of resolved molecules is

$$\Phi_{\mathrm{R}} = \bigcup_{i=1}^{n_{\mathrm{F}}} \Phi_{\mathrm{R}}^i \tag{27}$$

and the super-resolved image pattern is formed by taking the superimposition of resolution limited point patterns across the $n_{\mathrm{F}}$ frames, i.e.

$$\Phi_I = \bigcup_{i=1}^{n_{\mathrm{F}}} \Phi_I^i. \tag{28}$$

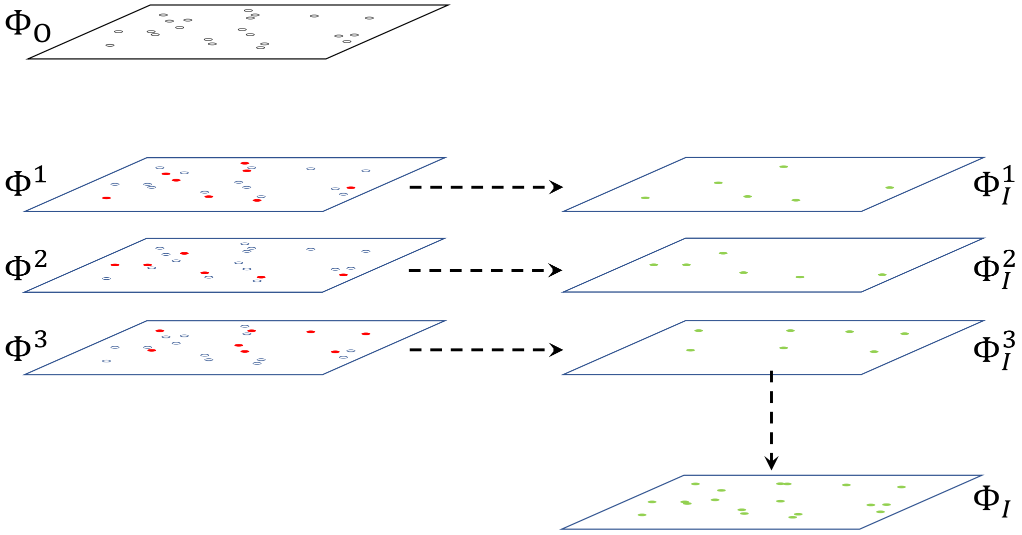


**Supplementary Figure 5.** Illustration of SMLM in which a pattern $\Phi_{\mathrm{O}}$ is imaged across 3 frames. Each frame is a thinned version of $\Phi_{\mathrm{O}}$ that is subject to an imaging operator with resolution limit $\alpha$. The resulting super-resolved image pattern $\Phi_I$ is the superimposition of individual frames.

The probabilistic resolution $p_{N_{\mathrm{O}},\alpha,q}$ of a super-resolved point process, now indicating its dependency on thinning rate $q$, is as defined in Definition 14, i.e. $\mathbb{P}(\mathbf{s} \in \Phi_{\mathrm{R}} | \mathbf{s} \in \Phi_{\mathrm{O}})$.

**Proposition 3.** *The probabilistic resolution $p_{N_{\mathrm{O}},\alpha,q}$ of a super-resolved process is equal to $p_{N_q,\alpha} = 1 - G_q(\alpha)$, where $G_q(\cdot)$ is the NND function for the process $N_q$, a classically $q$-thinned version of $N_{\mathrm{O}}$.*

*(A classically $q$-thinned version of $N_{\mathrm{O}}$ is where each event generated by $N_{\mathrm{O}}$ is independently kept with a probability $q$ or discarded with a probability $1-q$.)*

This result follows from the fact that the probability an event in $\Phi_{\mathrm{O}}$ is unaffected by resolution is the probability it is unaffected by resolution in the frame it appears in, i.e. $\mathbb{P}(\mathbf{s} \in \Phi_{\mathrm{R}} | \mathbf{s} \in \Phi_{\mathrm{O}}) = \mathbb{P}(\mathbf{s} \in \Phi_{\mathrm{R}}^i | \mathbf{s} \in \Phi^i)$, giving $p_{N_{\mathrm{O}},\alpha,q} = p_{N_q,\alpha}$. One corollary of this result is if one wishes to attain a minimum probabilistic

resolution of $p$ using an ARL of $\alpha$, then one must ensure $p \leq 1 - G_q(\alpha)$. The maximum value of $q$ required to achieve this is

$$q_{\max} = \max_q \{q \;:\; G_q(\alpha) \leq 1 - p\}. \tag{29}$$

We are able to verify the core idea behind SMLM procedures with the following result.

**Proposition 4.** *Let $N_{\mathrm{O}}$ be a spatial point process. Consider a single molecule localization microscopy experiment with assumptions 1-3 as outlined above. As the number of frames $n_{\mathrm{F}} \to \infty$ and $q \to 0$ such that $qn_{\mathrm{F}} = 1$, for $\mathbf{s} \in \Phi_{\mathrm{O}}$, $\mathbb{P}(\mathbf{s} \in \Phi_{\mathrm{R}}) \to 1$.*

This proposition states that as we tend the thinning of our pattern to zero while increasing the number of frames we image across, we tend to perfect probabilistic resolution, i.e. we guarantee that no event will be affected by resolution. The proof of this is found in Supplementary Note 12.4.

We now make use of this probabilistic reasoning about resolution to define the super-resolution limit for a point process.

**Definition 15.** *For a point process $N_{\mathrm{O}}$ imaged across $n_{\mathrm{F}} = q^{-1}$ frames subject to an algorithmic resolution limit $\alpha$, the super-resolution limit $\varsigma_{N_{\mathrm{O}},\alpha,q}$ is the $\alpha^*$ that solves*

$$p_{N_{\mathrm{O}},\alpha,q} = p_{N_{\mathrm{O}},\alpha^*}. \tag{30}$$

In other words, if $N_{\mathrm{O}}$ was imaged in a single frame, one would require a resolution limit of $\varsigma_{N_{\mathrm{O}},\alpha,q}$ to achieve the same probabilistic resolution as under the SMLM procedure with thinning rate $q$ and ARL $\alpha$.

**Proposition 5.** *The probabilistic super-resolution limit is $\varsigma_{N_{\mathrm{O}},\alpha,q} = G_{\mathrm{O}}^{-1}(G_q(\alpha))$.*

This follows directly from Propositions 2 and 3 and Definition 15.

## Supplementary Note 8: Computing probabilistic resolution

Probabilistic resolution and the super-resolution limit can not be estimated directly from images and must instead be computed for specific scenarios of interest. For example, one might ask: given I want to image a point pattern of this type, what conditions do I need to obtain a certain probabilistic resolution. Such computations require knowledge of the NND function for the data generating process of interest. The NND function is only analytically tractable for a very small set of idealized examples, and therefore so is the probabilistic resolution and super-resolution limits. We begin by looking at three such examples. We will then show how it can be estimated through simulations for more general cases.

## 8.1 Homogeneous Poisson Process

For an HPP $N_{\mathrm{O}}$ with intensity $\lambda_{\mathrm{O}}$, the NND distribution function is $G_{\mathrm{O}}(r) = 1 - \exp(-\lambda_{\mathrm{O}}\pi r^2)$. An HPP imaged with ARL $\alpha$ therefore has a probabilistic resolution $p_{N_{\mathrm{O}},\alpha} = \exp(-\lambda_{\mathrm{O}}\pi\alpha^2)$. For example, an HPP with $\lambda = 1$ events per μm$^2$ subject to ARL of $\alpha = 0.5$μm has $p_{N_{\mathrm{O}},\alpha} = 0.456$.

With regards to an SMLM experiment, for HPP $N_{\mathrm{O}}$ with intensity $\lambda_{\mathrm{O}}$, thinned process $N_q$ is an HPP with intensity $q\lambda_{\mathrm{O}}$. In this circumstance, the probabilistic resolution is $p_{N_{\mathrm{O}},\alpha,q} = \exp(-q\lambda_{\mathrm{O}}\pi\alpha^2)$, and from Proposition 5 the super-resolution limit is $\varsigma_{N_{\mathrm{O}},\alpha,q} = \sqrt{q}\alpha$. Further to this, Supplementary Equation (29) informs us that to ensure a probabilistic resolution of at least $p$ one requires $q \leq -(\lambda_{\mathrm{O}}\pi\alpha^2)^{-1}\ln p$. For example, suppose we image an HPP with intensity $\lambda_{\mathrm{O}} = 50$μm$^{-2}$ under an algorithmic resolution $\alpha = 0.5$μm, then $p_{N_{\mathrm{O}},0.5} = 8.8165 \times 10^{-8}$. Suppose we now use an SMLM imaging technique in which we image the HPP over 2000 frames, i.e. $q = 5 \times 10^{-4}$, then the probabilistic resolution increases to $p_{N_{\mathrm{O}},0.5,5\times10^{-4}} = 0.9806$ and the super-resolution limit is $\varsigma_{N_{\mathrm{O}},0.5,5\times10^{-4}} = 0.0112$μm, i.e. the probability an event is affected by resolution is the same as if $N_{\mathrm{O}}$ was imaged with an ARL of $0.0112$μm. Suppose we wish to achieve a probabilistic resolution of at least 0.99, then we would require $q \leq 2.56 \times 10^{-4}$.

## 8.2 Cluster Process

We now look at the case where $N_{\mathrm{O}}$ is a cluster process. The specific type of cluster process we consider is a Thomas process. This is a process in which parent events are generated according to an HPP with intensity $\kappa$, and each parent has a random number of offspring which is Poisson($\zeta$) distributed. These offspring are randomly placed around the parent according to a circular Gaussian distribution centred at the parent with covariance $\sigma^2 I$. Process $N_{\mathrm{O}}$ is comprised only of the offspring events. We note that the Thomas process is a type of stationary Cox process (Cressie, 1993, p. 663) and therefore the NND, as defined in Definition 7 is valid here. The NND function is given by Afshang et al. (2016) as

$$G_{\mathrm{O}}(r) = 1 - (1 - H(r)) \int_0^\infty \exp\left(-\zeta\left(1 - Q_1\left(\frac{v_0}{\sigma}, \frac{r}{\sigma}\right)\right)\right) f_{V_0}(v_0)\mathrm{d}v_0, \tag{31}$$

where

$$H(r) = 1 - \exp\left(-2\pi\kappa \int_0^\infty \left(1 - \exp\left(-\zeta\left(1 - Q_1\left(\frac{v}{\sigma}, \frac{r}{\sigma}\right)\right)\right) v\mathrm{d}v\right)\right), \tag{32}$$

$Q_1(\alpha, \beta)$ is the first-order Marcum $Q$-function defined as $Q_1(\alpha,\beta) = \int_\beta^\infty y\exp(-(y^2+\alpha^2)/2)I_0(\alpha y)\mathrm{d}y$ where $I_0(\cdot)$ is the 0th order modified Bessel function, and $f_{V_0}(v_0) = \frac{v_0}{\sigma^2}\exp(-v_0^2/2\sigma^2)$. For a given resolution limit $\alpha$ and process parameters $\kappa$, $\zeta$ and $\sigma$, the NDD function $G_{\mathrm{O}}(\alpha)$, and hence $p_{N_{\mathrm{O}},\alpha}$, can be computed through numerical integration.

For example, consider imaging a Thomas cluster process with a density of 10 clusters per $30$μm$^2$,

an expected 100 events per cluster and the events in each cluster distributed with a covariance matrix equal to $\sigma^2 I_2$, $\sigma = 0.05\mu$m (a typical simulation study from Rubin-Delanchy et al. (2015); Griffié et al. (2017) - see Supplementary Figure 6A for an example realization). For an ARL of $\alpha = 0.5\mu$m we have $G_{\text{O}}(\alpha) = 1$ to machine precision, which in turn gives a probabilistic resolution of $p_{N_{\text{O}},0.5} = 0$ to machine precision. Suppose we now image it using an SMLM procedure with $q = 1 \times 10^{-3}$. The $q$-thinned process can now be considered a Thomas cluster process with the same cluster density but with an expected $0.1$ events per cluster. This gives a probabilistic resolution of $p_{N_{\text{O}},0.5,1\times10^{-3}} = 0.882$ (the value of the curve at $q = 1 \times 10^{-3}$ in Supplementary Figure 6B) and a super-resolution limit of $3.56 \times 10^{-3}\mu$m. This calculation is illustrated in Supplementary Figure 6C.

Supplementary Equation (31) can further be used to numerically compute a bound on $q$ to attain a certain level of probabilistic resolution. For example, suppose we wish to achieve a probabilistic resolution of at least 0.99 we require $q \leq 7.97 \times 10^{-5}$.

### 8.3 Matérn I Hardcore Process

Suppose $N_{\text{O}}$ is a Matérn I hardcore process (Matérn, 1960) with hardcore distance greater than $\alpha$, i.e. no two events will be within $\alpha$ of each other with probability 1. This implies NND function $G_{\text{O}}(\alpha) = 0$ and therefore a probabilistic resolution of $p_{N_{\text{O}},\alpha} = 1$. Under an SMLM procedure the probabilistic resolution will continue to be 1 for any $q$, and therefore the super-resolution limit is 0 for all $q$. This equates to the pattern being perfectly resolvable.

Now suppose the hardcore distance is less than $\alpha$. An expression for the NND function is given in Al-Hourani et al. (2016). Due to it's complexity, we elect not to include it here. However, interested readers should follow the same procedure used in Supplementary Note 8.2 for the Thomas cluster process to compute quantities of interest.

### 8.4 Estimating the NND function and probabilistic resolution

The above examples have tractable expressions for the NND function from which the probabilistic resolution and super-resolution limit can be calculated. Expressions for the NND function quickly becomes intractable as we move to more complex point process models. Here, we illustrate how we can estimate probabilistic resolution and super-resolution limits under such circumstances.

The NND function of a point process can be estimated from a realized point pattern $\{\mathbf{s}_1, ..., \mathbf{s}_n\}$ on some region of interest $X \subset \mathbb{R}^2$. To do so, we use the following estimator from (Cressie, 1993, p. 614), there called the $\hat{G}_3$ estimator:

$$\hat{G}(r) = \frac{\sum_{i=1}^{n} I(r_{i,X} \leq r, d_i > r)}{\sum_{i=1}^{n} I(d_i > r)}, \qquad r > 0. \tag{33}$$

Here, $r_{i,X}$ and $d_i$ denote the distance from the $i$th event to, respectively, the nearest event in $X$ and the nearest boundary of $X$, and $I(B)$ is the indicator function of event $B$: $I(B) = 1$ if $B$ is true and 0 otherwise.

The procedure for computing $p_{N_{\mathrm{O}},\alpha,q}$ for a point-process $N_{\mathrm{O}}$ of interest for a given $\alpha$ and $q$ therefore proceeds as follows.

1. Simulate $\Phi_{\mathrm{O}} = \{\mathbf{s}_1, ..., \mathbf{s}_n\}$.
2. Compute $\hat{G}_{\mathrm{O}}(\alpha)$ from $\Phi_{\mathrm{O}}$ using Supplementary Equation (33). $1 - \hat{G}_{\mathrm{O}}(\alpha)$ provides an estimate of $p_{N_{\mathrm{O}},\alpha}$, the probabilistic resolution without an SMLM procedure.
3. For a particular $q$, randomly partition the events $\Phi_{\mathrm{O}}$ into $n_{\mathrm{F}} = \lfloor q^{-1} \rfloor$ thinned patterns $\Phi^1, ..., \Phi^{n_{\mathrm{F}}}$.
4. For each $\Phi^i$, $i = 1, ..., n_{\mathrm{F}}$, compute $\hat{G}^i_q(\alpha)$ using (33).
5. Compute the estimator for $G_q(\alpha)$ as

$$\hat{G}_q(\alpha) = n_{\mathrm{F}}^{-1} \sum_{i=1}^{n_{\mathrm{F}}} \hat{G}^i_q(\alpha). \tag{34}$$

   The probabilistic resolution $p_{N_{\mathrm{O}},\alpha,q}$ is estimated as $1 - \hat{G}_q(\alpha)$.

6. The super-resolution limit can be estimated by computing $\hat{G}_{\mathrm{O}}(r)$ over a range of $r$ and numerically finding $\hat{G}_{\mathrm{O}}^{-1}(\hat{G}_q(\alpha))$.

We illustrate this with an example.

**Example: Tubulin**

We consider a tubulin localization data set. We assume in a typical imaging experiment that the position, curvature and length of the microtubules are random and hence can be modelled as a fibre process (Stoyan et al., 1995, Chapter 9), and that the tubulin locations are realized on the fibres according to a Poisson process. In other words, if $S_i \subset \mathbb{R}^2$ is a fibre (microtubule) and $S = \bigcup_{i=1} S_i \subset \mathbb{R}^2$ the fibre process (collection of microtubules), then the tubulin process is Cox with random intensity $\Lambda(\mathrm{s}) = \omega \cdot \mathbb{1}_S(\mathrm{s})$, $\omega > 0$. Provided this fibre process is stationary (Stoyan et al., 1995, p. 282 ), the tubulin point process will be a stationary Cox process (Stoyan et al., 1995, p. 156 ), and hence has a NND function that can be estimated from a realization. The dataset we consider here is taken from the Single Molecule Localization Microscopy Symposium 2013 data challenge (Sage et al., 2015) and consists of 100000 emitters over a 40µm × 40µm region of interest, thus giving an estimated intensity of the object process of $\hat{\lambda}_{\mathrm{O}} = 62.5$µm$^{-2}$ (see Supplementary Figure 6D). The structure is imaged over 2401 frames, giving $q = 1/2401 \approx 4.90 \times 10^{-4}$

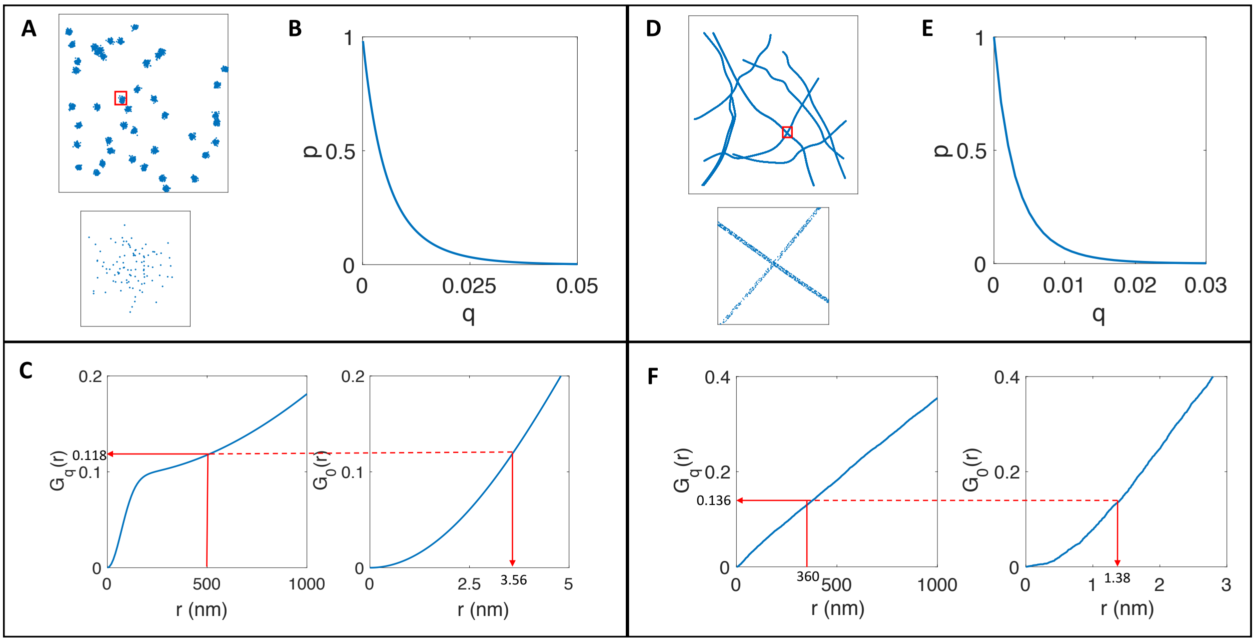


**Supplementary Figure 6.** **A.** A realization of a Thomas Cluster process with an expected 10 clusters per $30\mu m^2$ and 100 expected events per cluster spatially distributed according to a Gaussian distribution with standard deviation $50nm$ (a typical simulation study from Rubin-Delanchy et al. (2015); Griffié et al. (2017)). **B.** A curve showing how the probabilistic resolution of the process changes with thinning rate $q$ for $\alpha = 0.5\mu m$. **C.** Demonstration of how the super-resolution limit can be computed from the NND function when $q = 0.001$ and $\alpha = 0.5\mu m$ – see text for details. **D.** Tubulin data from the Single Molecule Localization Microscopy Symposium 2013 data challenge (Sage et al., 2015). **E.** A curve showing how the probabilistic resolution of the process changes with thinning rate $q$ for $\alpha = 0.360\mu m$. **F.** Demonstration of how the super-resolution limit can be computed from the NND function when $q = 1/2401$ and $\alpha = 0.360\mu m$ – see text for details.

and an average of 41.6 emitters per frame. The data set conforms to assumptions 1-3 of Supplementary Note 7.

With an ARL of $0.360\mu m$ (the estimated ARL of Algorithm 2 - see Figure 2), the probabilistic resolution is estimated to be $p_{N_O,0.360,4.9e-4} \approx 0.864$ (the value of the curve at $q = 1/2401$ in Supplementary Figure 6E). This leads to a super-resolution limit of $1.38 \times 10^{-3}\mu m$, i.e. with just a single image of the entire object pattern one would need an algorithmic resolution limit of $1.38 \times 10^{-3}\mu m$ to achieve the same probabilistic resolution as the SMLM procedure. This calculation is illustrated in Supplementary Figure 6F.

## Supplementary Note 9: Estimating the algorithmic resolution limit

We acknowledge that there may be several suitable methods for estimating the ARL as defined in Definition 12. Here, we provide one method and show that under certain attainable conditions it has preferable statistical properties. The method we present makes use of the pair correlation function to form an estimator for the algorithmic correlation radius $\rho$, defined in Definition 11. From Supplementary Equation 15, the estimator for the ARL is then given as $\hat{\alpha} = \hat{\rho}/2$.

We begin by modelling the pair correlation function for the image process as

$$g_I(r) = \begin{cases} h(r) & 0 \leq r < \rho \\ 1 & r \geq \rho, \end{cases} \tag{35}$$

where there exists an $0 \leq s < \rho$ such that $h(r)$ is not a constant 1 on $(s, \rho)$. Suppose we construct an unbiased estimator $\hat{g}_I(r)$ of $g_I(r)$ (see the Image Analysis section in the *Methods* for details) and compute it on each element of $\mathcal{R} = \{r_1, ..., r_m\}$, a finely, regularly spaced collection of proposals for $\rho$. We assume $\rho \in \mathcal{R}$, that the largest element $r_m$ is certainly larger than $\rho$ and smallest element $r_1$ is certainly smaller than $\rho$. We further assume that $\hat{g}_I(r_i) = g_I(r_i) + \epsilon_i$ at $r_i \in \mathcal{R}$, where $\epsilon_1, ..., \epsilon_m$ are zero mean, iid random variables with finite variance $\sigma^2 > 0$.

The estimator of $\rho$ we provide is defined as

$$\hat{\rho} = \underset{r_i \in \mathcal{R}}{\operatorname{argmin}}\, T(r_i) \tag{36}$$

where

$$T(r_i) = (m - i + 1)^{-1}(m - i)^{-1} \sum_{j=i}^{m} (\hat{g}_I(r_j) - \bar{g}_i)^2 \tag{37}$$

and $\bar{g}_i = (m - i + 1)^{-1} \sum_{j=i}^{m} \hat{g}_I(r_j)$. To show that this is estimating $\rho$, as required, we consider $E\{T(r_i)\}$.

The intuition to this is that the function $T(r_i)$ is the standard error of the pair correlation estimator across all proposals to the right of $r_i$, including $r_i$ itself. Therefore, moving from right to left, while $g_I$ remains constant $T$ will decrease in value. Once $g_I$ starts to deviate from the constant value of one, provided it does so by a certain amount, $T$ will start to increase in value; leaving the minimum of $T$ to be at the proposal closest to $\rho$. In practice, this means this estimator will be conservative as it will choose the first point from the right that appears to deviate significantly from one.

To formulate this, let $\mathcal{R} = \{r_1, ..., r_{K-1}, \rho, r_{K+1}, ..., r_m\}$ be the collection of proposals indicating that the true, unknown value of $\rho$ is $r_K$. For $i < K$ we let $n_{\mathrm{L},i} = K - i$, for $K \leq i < m$ we let $n_{\mathrm{R},i} = m - K + 1$, and for $i < K$ we let $n_i = n_{\mathrm{L},i} + n_{\mathrm{R},K}$. Subscripts $\mathrm{L}$ and $\mathrm{R}$ indicate a partitioning of the proposals $r_i, ..., r_m$ that fall to the left or right of $r_K$, respectively. With $N_i = n_i^2 - n_i$, $N_{\mathrm{L},i} = n_{\mathrm{L},i}^2 - n_{\mathrm{L},i}$ and $N_{\mathrm{R},i} = n_{\mathrm{R},i}^2 - n_{\mathrm{R},i}$, the function $T(r_i)$ can be written as (Baker and Nissim, 1963)

$$T(r_i) = \begin{cases} \frac{N_{\mathrm{L},i}}{N_i} s_{\mathrm{L},i}^2 + \frac{N_{\mathrm{R},K}}{N_i} s_{\mathrm{R},K}^2 + \frac{n_{\mathrm{L},i} n_{\mathrm{R},K} (\bar{g}_{\mathrm{L},i} - \bar{g}_{\mathrm{R},K})^2}{n_i N_i} & 1 \leq i < K \\ \frac{1}{n_{\mathrm{R},i}} s_{\mathrm{R},i}^2 & K \leq i < m \end{cases}, \tag{38}$$

where

$$\bar{g}_{\mathrm{L},i} = \frac{1}{n_{\mathrm{L},i}} \sum_{j=i}^{K-1} \hat{g}_I(r_j) \qquad 1 \leq i < K \tag{39}$$

$$\bar{g}_{\mathrm{R},i} = \frac{1}{n_{\mathrm{R},i}} \sum_{j=i}^{m} \hat{g}_I(r_j) \qquad K \leq i < m \tag{40}$$

$$s^2_{\mathrm{L},i} = \frac{1}{n_{\mathrm{L},i} - 1} \sum_{j=i}^{K-1} \left(\hat{g}_I(r_j) - \bar{g}_{\mathrm{L},i}\right)^2 \qquad 1 \leq i < K \tag{41}$$

$$s^2_{\mathrm{R},i} = \frac{1}{n_{\mathrm{R},i} - 1} \sum_{j=i}^{m} \left(\hat{g}_I(r_j) - \bar{g}_{\mathrm{R},i}\right)^2 \qquad K \leq i < m. \tag{42}$$

Taking the expected value, it follows that

$$E\{T(r_i)\} = \begin{cases} \frac{N_{\mathrm{L},i}}{N_i n_{\mathrm{L},i}}(\sigma^2 + \kappa_i^2) + \frac{N_{\mathrm{R},K}}{N_i n_{\mathrm{R},K}}\sigma^2 + \frac{n_{\mathrm{L},i} n_{\mathrm{R},K} E\{(\bar{g}_{\mathrm{L},i} - \bar{g}_{\mathrm{R},K})^2\}}{n_i N_i} & 1 \leq i < K \\ \frac{1}{n_{\mathrm{R},i}}\sigma^2 & K \leq i < m, \end{cases} \tag{43}$$

where

$$\kappa_{\mathrm{L},i} = \frac{1}{n_{\mathrm{L},i}} \sum_{j=i}^{K-1} \left(g(r_i) - \mu_{\mathrm{L},i}\right)^2 \tag{44}$$

and

$$\mu_{\mathrm{L},i} = \frac{1}{n_{\mathrm{L},i}} \sum_{j=i}^{K-1} g(r_i). \tag{45}$$

To demonstrate that this is a suitable estimator for the radius of correlation, we look to find a sufficient condition for

$$\operatorname*{argmin}_{r_i \in \mathcal{R}} E\{T(r_i)\} = r_K = \rho, \tag{46}$$

i.e. the minimum of the expected value of the function is at $\rho$. Given $E\{T(r_i)\} < E\{T(r_j)\}$ for all $K \leq i < j < n$, such a condition needs to ensure $E\{T(r_i)\} > E\{T(r_K)\}$ for all $1 \leq i < K$. We begin by considering a necessary and sufficient condition for $E\{T(r_{K-1})\} > E\{T(r_K)\}$. For $i = K - 1$, we have $n_{1,i} = 1$ and $N_1 = 0$, giving

$$\frac{N_{\mathrm{R},K}}{N_i n_{\mathrm{R},K}}\sigma^2 + \frac{n_{1,i} n_{\mathrm{R},K} E\{(\bar{g}_{1,i} - \bar{g}_{\mathrm{R},K})^2\}}{n_i N_i} > \frac{1}{n_{\mathrm{R},K}}\sigma^2 \tag{47}$$

is necessary and sufficient for $E\{T(r_{K-1})\} > E\{T(r_K)\}$. In this circumstance, $\bar{g}_{1,i} = \hat{g}(r_{K-1})$ and it follows (through manipulation) that (47) holds if and only if

$$(g(r_{K-1}) - 1)^2 > \frac{n_{\mathrm{R},K} + 1}{n_{\mathrm{R},K}}\sigma^2. \tag{48}$$

This states that provided $g$ deviates away from 1 by a larger amount than $((n_{\mathrm{R},K}+1)/n_{\mathrm{R},K})^{-1/2}\sigma$ (which is approximately equal to $\sigma$ for large $n_{\mathrm{R},K}$) then $E\{T(r_{K-1})\} > E\{T(r_K)\}$, i.e. there exists a local minimum at $r_K$. For it to be a global minimum it is sufficient to have $E\{T(r_i)\} > E\{T(r_K)\}$ for all $i < K$. Further manipulation can be used to show that a sufficient condition for $\underset{r_i \in \mathcal{R}}{\operatorname{argmin}}\, E\{T(r_i)\} = \rho$ is

$$\frac{(n_{\mathrm{L},i}-1)n_{\mathrm{R},K}}{N_{\mathrm{L},i}+n_{\mathrm{L},i}n_{\mathrm{R},K}}\kappa_i^2 + \frac{n_{\mathrm{L},i}n_{\mathrm{R},K}^2}{n(N_{\mathrm{L},i}+n_{\mathrm{L},i}n_{\mathrm{R},K})}(\mu_{\mathrm{L},i}-1)^2 > \sigma^2 \qquad \text{for all } 1 \le i < K. \tag{49}$$

Suppose we are able, through simulation, to compute $M$ independent and identically distributed estimates of the pair correlation function $\hat{g}_{I,1}(r_i), \hat{g}_{I,2}(r_i), ..., \hat{g}_{I,M}(r_i)$, for all $r_i \in \mathcal{R}$, each with error of variance $\sigma^2$, then estimator $\hat{g}_I(r_i) = \sum_{j=1}^{M} g_{I,j}(r_i)$ has variance $\sigma^2/M$. Therefore, provided we choose an $M$ such that

$$M > \left(\frac{(n_{\mathrm{L},i}-1)n_{\mathrm{R},K}}{N_{\mathrm{L},i}+n_{\mathrm{L},i}n_{\mathrm{R},K}}\kappa_i^2 + \frac{n_{\mathrm{L},i}n_{\mathrm{R},K}^2}{n(N_{\mathrm{L},i}+n_{\mathrm{L},i}n_{\mathrm{R},K})}(\mu_{\mathrm{L},i}-1)^2\right)^{-1} \sigma^2 \qquad \text{for all } 1 \le i < K, \tag{50}$$

we will satisfy sufficient condition (49).

## 9.1 Verifying it is a true change point

We propose a test to verify whether an estimate of a correlation radius, as defined in (36) and (37), is due to a true change point in the pair correlation function. Suppose a genuine radius of correlation $\rho > 0$ does exist, then with everything to the right of $\rho$ being a constant, we should find the variance of $\hat{g}_I(r_K), ..., \hat{g}_I(r_m)$ to be smaller than the variance of $\hat{g}_I(r_1), ..., \hat{g}_I(r_{K-1})$. We propose an $F$-test to check whether the difference in variance is statistically significant.

Let $s^2_{\mathrm{L},K}$ be the variance of $\hat{g}_I(r_1), \hat{g}_I(r_2), ..., \hat{g}_I(r_{K-1})$ and $s^2_{\mathrm{R},K}$ be the variance of $\hat{g}_I(r_K), ..., \hat{g}_I(r_m)$, defined by (41) and (42), respectively. We look to test the null hypothesis $H_0$ that states $s^2_{\mathrm{L},K} = s^2_{\mathrm{R},K}$. Rejecting $H_0$ in favour of the accepting the alternative ($s^2_{\mathrm{L},K} \neq s^2_{\mathrm{R},K}$) is equivalent to accepting there is a genuine change point in the pair correlation function at $\hat{\rho}$.

Under the null hypothesis

$$\frac{s^2_{\mathrm{L},K}}{s^2_{\mathrm{R},K}} \sim F_{n_{\mathrm{L},K}-1, n_{\mathrm{R},K}-1} \tag{51}$$

where $F_{n_{\mathrm{L},K}-1, n_{\mathrm{R},K}-1}$ represents the $F$ distribution with $n_{\mathrm{L},K}-1$ and $n_{\mathrm{R},K}-1$ degrees of freedom. We therefore reject the null hypothesis at the $p$ significance level when $\frac{s^2_{\mathrm{L},K}}{s^2_{\mathrm{R},K}} > F^p_{n_{\mathrm{L},K}-1, n_{\mathrm{R},K}-1}$, where $F^p_{n_{\mathrm{L},K}-1, n_{\mathrm{R},K}-1}$ denotes the upper $p \cdot 100\%$ of the $F_{n_{\mathrm{L},K}-1, n_{\mathrm{R},K}-1}$ distribution.

### 9.2 Bootstrapping intervals of confidence

Consider the case where we have formed $\hat{g}_I(r_i)$, $r_i \in \mathcal{R}$, by averaging $M$ independent and identically distributed estimators $\hat{g}_{I,1}(r_i), ..., \hat{g}_{I,M}(r_i)$. We can place all estimates in the $m \times M$ matrix

$$G = \begin{pmatrix} \hat{g}_{I,1}(r_1) & \cdots & \hat{g}_{I,M}(r_1) \\ \vdots & & \vdots \\ g_{I,1}(r_m) & \cdots & \hat{g}_{I,M}(r_m) \end{pmatrix}. \tag{52}$$

Using the resampling with replacement framework of Efron and Tibshirani (1993), one can create $R$ bootstrapped estimates $\hat{g}_I^{*1}(r_i), \hat{g}_I^{*2}(r_i), ..., \hat{g}_I^{*R}(r_i)$ by creating $m \times M$ matrices $G^{*1}, G^{*2}, ..., G^{*R}$, where the $M$ columns of each $G^{*k}$, $k = 1, .., R$ are $M$ randomly sampled (with replacement) columns of $G$. Bootstrap estimate $\hat{g}_I^{*k}(r_i)$ is then computed by averaging across the columns,

$$\hat{g}_I^{*k}(r_i) = \sum_{j=1}^{M} (G^{*k})_{ij}. \tag{53}$$

We then implement the estimator of the radius of correlation described above on each of the $R$ pair correlation estimates $[\hat{g}_I^{*k}(r_1), ..., \hat{g}_I^{*k}(r_m)]^T$, $k = 1, ..., R$, to produce bootstrapped estimates $\hat{\alpha}^{*1}, ..., \hat{\alpha}^{*R}$. For $0.5 < p < 1$, letting $\hat{\alpha}^{*(p)}$ and $\hat{\alpha}^{*(1-p)}$ be the $100 \cdot p$th and $100 \cdot (1-p)$th empirical percentiles from $\hat{\alpha}^{*1}, \hat{\alpha}^{*2}, \ldots, \hat{\alpha}^{*R}$, a percentile bootstrap interval of length $1 - 2p$ is given by

$$[\hat{\alpha}_{\%,\text{lo}}, \hat{\alpha}_{\%,\text{up}}] \approx [\hat{\alpha}^{*(p)}, \hat{\alpha}^{*(1-p)}]. \tag{54}$$

### 9.3 Example

We have applied the estimator and bootstrapping procedure presented here to Algorithms 1, 2 and 3. The vector of proposals used was $\mathcal{R} = [0.020, 0.025, 0.030, ..., 2.000]^T$ and $M = 2000$ independent estimates of the pair correlation function were computed. These were averaged together to form $\hat{g}_I$, from which $\hat{\alpha}$ is estimated. A further 10000 bootstrapped estimates were computed via the procedure in Supplementary Note 9.2. Details on the simulations and analysis can be found in the *Methods*. The results are shown in Table 1.

Further insight into issues encountered when estimating $\alpha$ can be found by looking at the histogram of the bootstrapped estimates. Algorithms 1 and 2 have a neat, unimodal distribution for $\hat{\alpha}$. This can be explained by observing that the pair correlation functions shown in Figure 2d of the main paper have a smooth shape that, moving from the left, cleanly settles on a constant value of one. This leaves little ambiguity as to the value of the radius of correlation, and thus the algorithmic resolution limit. However,

| Algorithm | $\hat{\alpha}$ (µm) | $[\hat{\alpha}^{*(10\%)}, \hat{\alpha}^{*(90\%)}]$ |
|---|---|---|
| Algorithm 1 | 0.3625 | [0.3325,0.4325] |
| Algorithm 2 | 0.3600 | [0.3550,0.5150] |
| Algorithm 3 | 0.6200 | [0.3775,0.6875] |

Table 1: The estimated algorithmic resolution limit and the 80% bootstrapped confidence interval for three different algorithms at a density/intensity of $\lambda_O = 1$ molecule per µm$^2$.

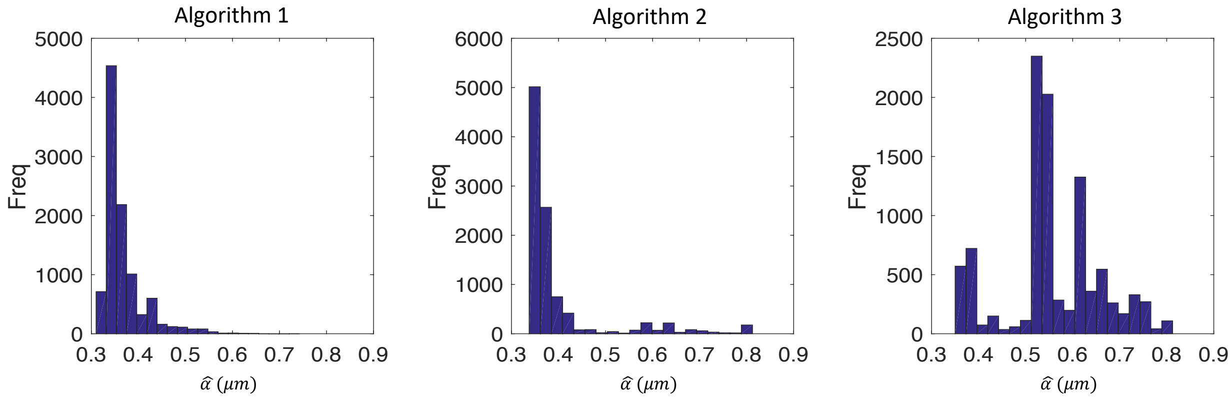


**Supplementary Figure 7.** Histogram of the bootstrapped estimates for $\hat{\alpha}$ for the Algorithm 1, 2 and 3 at a density/intensity of $\lambda_O = 1$ molecule per µm$^2$.

with Algorithm 3 we see the bootstrapped estimates of $\alpha$ take a multimodal distribution. This again can be understood from the pair correlation function in Figure 2d of the main paper in which there appears to be a interval over which the function approximately settles to one but with ambiguity over when one would consider it to have actually occurred.

# Supplementary Note 10: Further analysis

In this section we look at two further properties - molecule density and signal to noise ratio (SNR) - and analyze the effect they have on algorithmic resolution limit.

## 10.1 The effect of molecule density

We note that the definitions for the radius of correlation and algorithmic resolution limit, Definitions 11 and 12, respectively, do not depend on the intensity (also referred to as the density) $\lambda_O$ of the object process. Supplementary Figure 8 illustrates that this is what we observe in practice by demonstrating the pair correlation function for three different algorithms (imaging operators) has strikingly similar shape across a wide range of intensities. In particular, by eye, the point at which it settles to a constant one appears to be very similar for molecule densities (intensities) up to and including 1 molecule per µm$^2$. However, for two of the algorithms analyzed (Algorithms 1 and 2), there appears to be a break down of this

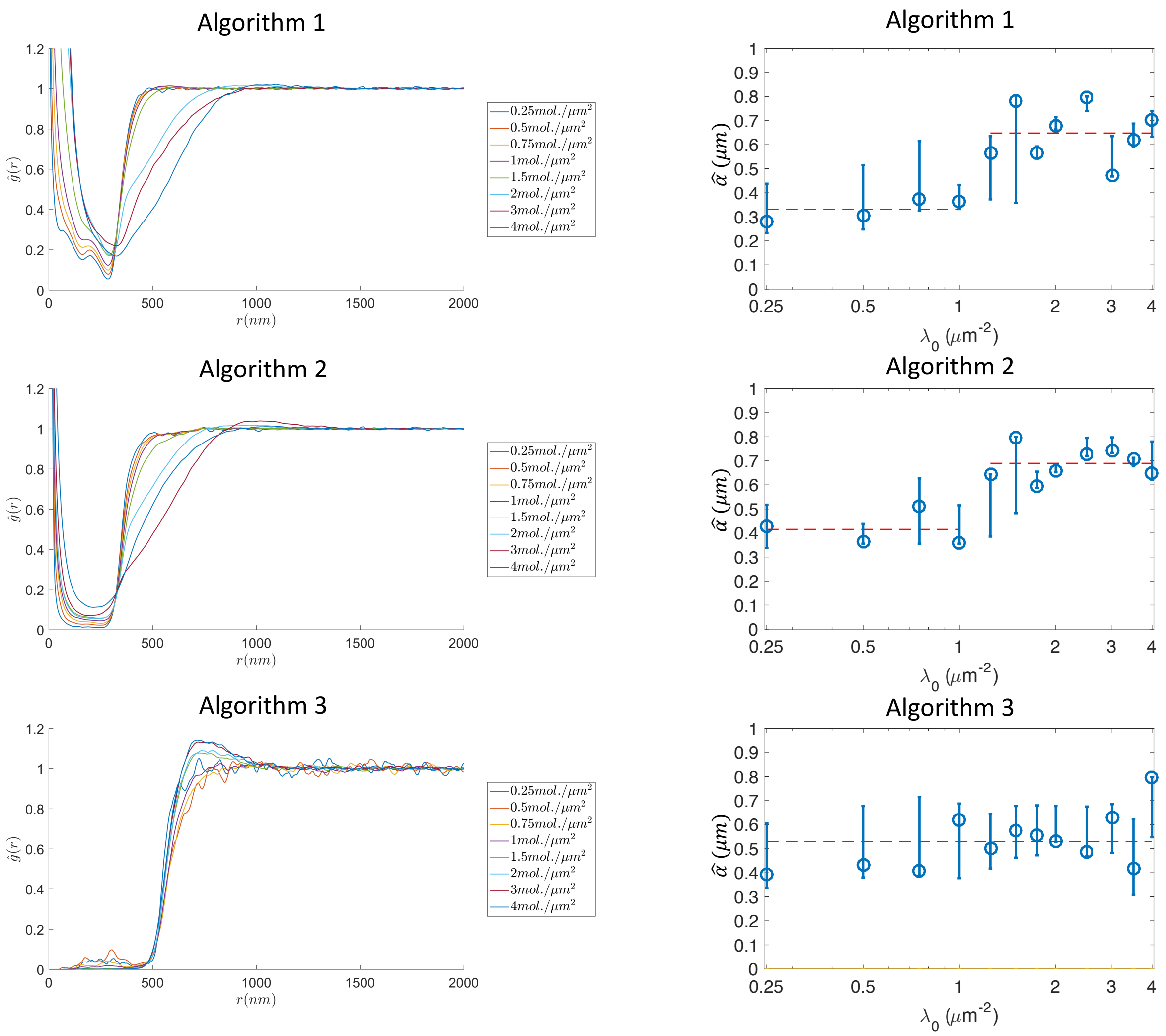


**Supplementary Figure 8.** Left: The estimated pair correlation function for a range of intensities (molecule densities) $\lambda_O$ for Algorithms 1, 2 and 3. See *Methods* for details on simulations and estimating the pair correlation functions. Right: The estimated algorithmic resolution limits (see Supplementary Note 9) for the pair correlation functions on the left. The bottom and top of the bars indicate the 10th and 90th percentile of the bootstrapped distribution for $\hat{\alpha}$, respectively. The red dotted line indicates the average estimate over the range it spans.

behavior for densities greater than 1 molecule per $\mu m^2$. This is, to some extent, verified by applying the estimator for $\alpha$ detailed in Supplementary Note 9, the results of which are shown on the right-hand-side of Supplementary Figure 8. This is not surprising; there will be a point at which the image becomes saturated and the algorithm is simply unable to perform its task. Interestingly, Algorithm 3 appears to have an approximately constant algorithmic resolution limit across all densities studied.

## 10.2 The effect of signal to noise ratio

We here explore the effect of SNR on the algorithmic resolution limit by changing the number of photons emitted from each molecule, which equates to varying the signal strength. Supplementary Figure 9 shows that above 100 photons per molecule the algorithmic resolution limit remains constant for Algorithms 1 and

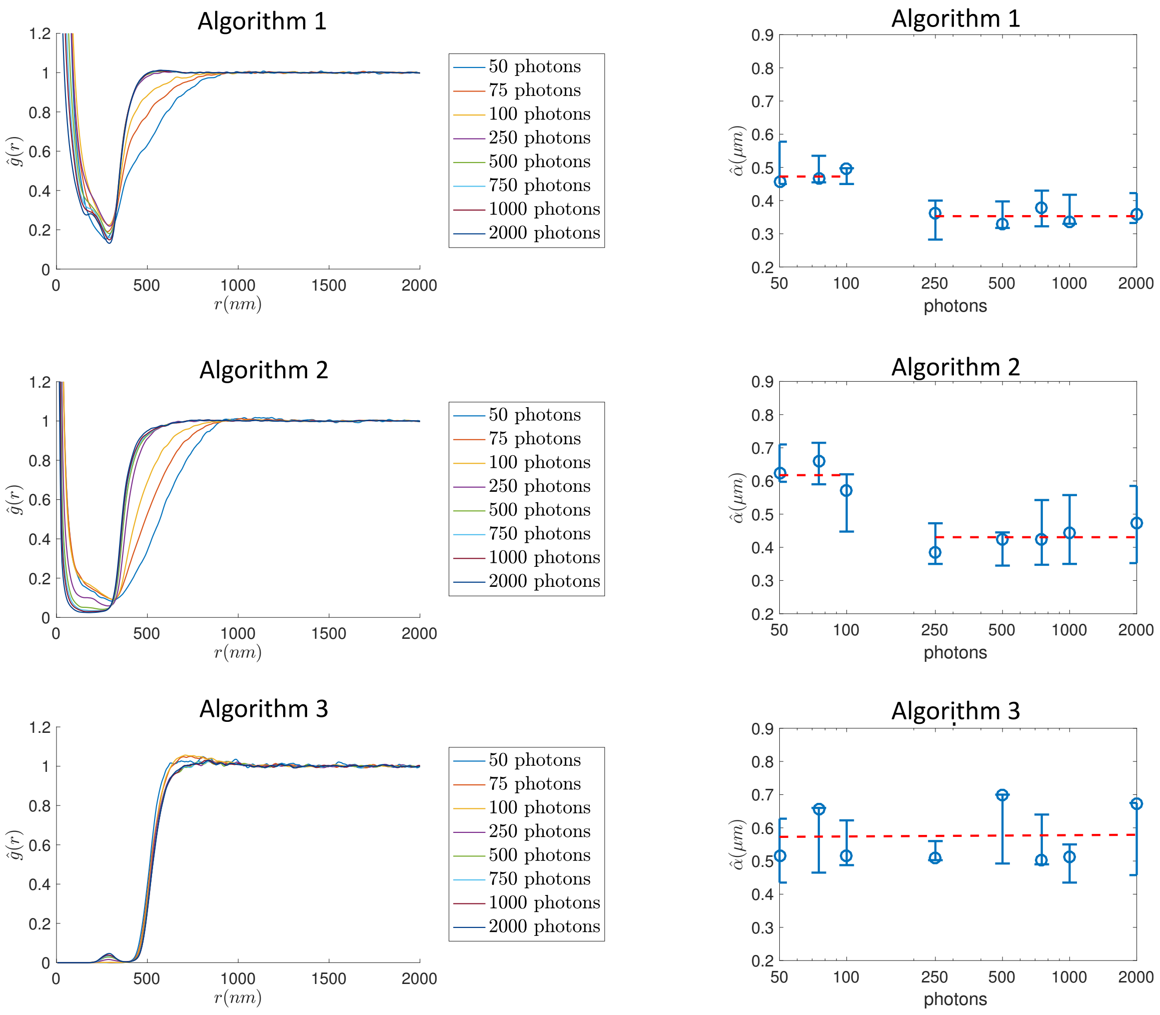


**Supplementary Figure 9.** Left: The estimated pair correlation function for a range of photon emission counts (signal strengths) for Algorithms 1, 2 and 3. See *Methods* for details on simulations and estimating the pair correlation functions. Right: The estimated algorithmic resolution limits (see Supplementary Note 9) for the pair correlation functions on the left. The bottom and top of the bars indicate the 10th and 90th percentile of the bootstrapped distribution for $\hat{\alpha}$, respectively. The red dotted line indicates the average estimate over the range it spans.

2. However for 100 photons and below there is an increase in the algorithmic resolution limit. This can be seen in both the values of the estimated algorithmic resolution limit and the shape of the pair-correlation function. This degradation at low SNR of the algorithms' ability to distinguish between objects is in keeping with the results of Ram et al. (2006) and further discussion on this is provided in the *Results* section of the main text. This behavior is not noticeable with Algorithm 3, however its algorithmic resolution limit in all cases is significantly above Rayleigh's limit and therefore it is unsurprising that the effect predicted by Ram et al. (2006) is undetectable.

## Supplementary Note 11: Analyzing resolution limited point patterns

The pair-correlation function $g(r)$ (see Definition 3) and Ripley's K-function $K(r)$ (see Definition 6) are the two most common functions characterizing point processes that are estimated from a realized point pattern.

The benefit of the pair correlation function is it gives a measure of correlation at a specific distance $r$ of interest, however, it can be difficult to estimate, particularly from a single realization with a low number of events. The $K$-function, on the other-hand, integrates $rg(r)$ over the range $[0, r]$, so smooths out the localized information in the pair-correlation function. However, estimating it is straight forward and well-studied (Illian et al., 2008; Ripley, 1981; Cressie, 1993; Lagache et al., 2013).

For analyzing resolution limited point processes, we suggest, where possible, the use of the pair-correlation function. As Supplementary Figure 3 shows, the effects of limited resolution in the correlation structure of the process occur over a distance of $2\alpha$, therefore $g(r)$ should only be used for analysis on distances greater than $2\alpha$. However, because the pair correlation function gives localized information on the process' correlation structure the resolution effect on $0 < r < 2\alpha$ does not propagate into $g$ for $r \geq 2\alpha$.

In contrast, due to the integration involved, the effects of limited resolution on the interval $(0, 2\alpha)$ propagates into the $K$-function at all $r$, as shown in Supplementary Figure 3C and 3D. For this reason we recommend the use of the $K_{2\alpha}$ function which we define as

$$K_{2\alpha}(r) \equiv 2\pi \int_{2\alpha}^{r} r' g(r') \mathrm{d}r' = K(r) - K(2\alpha) \qquad r > 2\alpha. \tag{55}$$

In the situation where $N_{\mathrm{O}}$ is an HPP and the image point process $N_I$ under analysis has second-order intensity of form given in Supplementary Equation (9), $K_{2\alpha} = \pi r^2 - 4\pi\alpha^2$, and hence if we define $L_{2\alpha}(r) \equiv \sqrt{(K_{2\alpha}(r) + 4\pi\alpha^2)/\pi}$ then $L_{2\alpha}(r) = r$ and $L_{2\alpha}(r) - r = 0$ under CSR of $N_{\mathrm{O}}$.

The function $K_{2\alpha}(r)$ can be estimated as $\hat{K}_{2\alpha}(r) = \hat{K}(r) - \hat{K}(2\alpha)$, $r > 2\alpha$, where $\hat{K}$ is the traditional $K$-function estimator of Ripley (1981)

$$\hat{K}(r) = \frac{A}{n(n-1)} \sum_{i=1}^{n} \sum_{j=1}^{n} w_{ij} I(0 < d_{ij} < r), \tag{56}$$

where $A$ denotes the area in the object space corresponding to the image being analyzed, $w_{ij}$ denotes the Ripley's isotropic edge correction weights Ripley (1981), $n$ denotes the total number of localizations, and

$d_{ij}$ denotes the distance between the $i^{th}$ and $j^{th}$ localizations. The indicator function is defined as

$$I(0 < d_{ij} < r) := \begin{cases} 1, & \text{if } 0 < d_{ij} < 1 \\ 0, & \text{otherwise.} \end{cases} \tag{57}$$

Further discussion is provided in the *Methods* section of the main text.

## 11.1 Ripley's $K$-function for SMLM data

When dealing with SMLM data, it is possible to make advantage of the temporal information encoded into the data to mitigate for the algorithmic resolution limit. Given realizations $\phi_I^1, \phi_I^2, ..., \phi_I^{n_\mathrm{F}}$ of the point patterns $\Phi_I^1, \Phi_I^2, ..., \Phi_I^{n_\mathrm{F}}$ for each of the $n_\mathrm{F}$ frames, we know that a pair of localizations that appear in separate frames cannot mutually interfere with each other through resolution issues. Typically, the classical estimator for Ripley's $K$-function would sequentially move through every event (localization) in the combined realization $\phi_I = \bigcup_{i=1}^{n_\mathrm{F}} \phi_I^i$ and count how many of the other events were within a distance $r$ of that event (with edge-corrections if needed). The counts are then averaged over the entire dataset to get an estimate for the expected number of events within a distance $r$ of an arbitrary event.

When estimating Ripley's $K$-function in the SMLM setting, one can use the following strategy: again, move through every event in $\phi_I$, however, when counting the number of events within a distance r, do not include events in the same frame and only include events from the other frames. This means no two events which may be mutually interfering with each other through resolution effects will be used together in the estimate, allowing estimation of the $K$-function for all $r \geq 0$.

Suppose the realized localizations in frame $i$ are $\phi_I^i = \{\mathbf{s}_1^i, ..., \mathbf{s}_{m_i}^i\}$, $i = 1, ..., n_\mathrm{F}$ then this estimator can be expressed as

$$\hat{K}(r) = \frac{A}{n} \sum_{i=1}^{n_\mathrm{F}} \frac{1}{m - m_i} \sum_{j=1}^{m_i} \sum_{\substack{k=1 \\ k \neq i}}^{n_\mathrm{F}} \sum_{j=1}^{m_k} w_{(ij)(kl)} I(0 < d_{(ij)(kl)} < r), \tag{58}$$

where $m_i = |\phi_I^i|$ is the number of localizations in frame $i$, $m$ is the total number of localizations across all frames, $w_{(ij)(kl)}$ is the edge correction weight for the localizations $\mathbf{s}_j^i$ and $\mathbf{s}_l^k$, and $d_{(ij)(kl)}$ is the Euclidean distance between localizations $\mathbf{s}_j^i$ and $\mathbf{s}_l^k$.

# Supplementary Note 12: Appendices

## 12.1 An alternative model for the imaging operator

Consider the following model for an imaging operator $I$.

**Model 3.** *Let $N_{\mathrm{O}}$ be a point process and let $\alpha > 0$ be a distance partitioning event set $\Phi_{\mathrm{O}}$ into event sets $\Phi_{\mathrm{R}}$ and $\Phi_{\mathrm{U}}$, such that resolved events $\Phi_{\mathrm{R}} \subseteq \Phi_{\mathrm{O}}$ are all the events in $\Phi_{\mathrm{O}}$ that have no other event within a distance $\alpha$ of it and unresolved events $\Phi_{\mathrm{U}}$ are all events in $\Phi_{\mathrm{O}}$ that has one of more events within distance $\alpha$ of it. Unresolved events $\Phi_{\mathrm{U}}$ can be further partitioned into resolution limited sub-patterns (see Definition 13) $\mathcal{S}_1, ..., \mathcal{S}_{M(\alpha)}$ such that $\Phi_{\mathrm{U}} = \bigcup_{i=1}^{M(\alpha)} \mathcal{S}_i$ and $\mathcal{S}_i \cap \mathcal{S}_j$ for all $i \neq j$, $i, j = 1, ..., M(\alpha)$. The image event set $\Phi_I$ is constructed as*

$$\Phi_I = \Phi_{\mathrm{R}} \cup \left( \bigcup_{i=1}^{M(\alpha)} \bar{s}_i \right) \tag{59}$$

*where $\bar{s}_i$ is the mean position of RSLP $\mathcal{S}_i = \{\mathbf{s}_{i,1}, \mathbf{s}_{i,2}, ...\}$, $i = 1, 2, ...$, i.e.*

$$\bar{s}_i = \frac{1}{|\mathcal{S}_i|} \sum_{j=1}^{|\mathcal{S}_i|} \mathbf{s}_{i,j}. \tag{60}$$

Under this model, all events from $N_{\mathrm{O}}$ with no other event within a distance $\alpha$ of it are resolved into the image process $N_I$. Events that have another event within a distance $\alpha$ of it, i.e. belong to an RSLP, are not resolved into $N_I$. Instead, the average position of the RSLP becomes an event in $N_I$. This modeling assumption appears to be a reasonable approximation to the type of behavior seen in the images of Supplementary Figure 3E.

While the second order properties of the image process $N_I$ for HPP $N_{\mathrm{O}}$ appear intractable, we are able to simulate these processes and estimate the pair correlation function and radius of correlation using the method presented in Section 9. In Supplementary Figure 10 is presented the estimated pair correlation function and estimated radius of correlation under this model, demonstrating the radius of correlation $\rho$ is indeed estimated to be at around $2\alpha$, and thus $\rho/2$ is an appropriate definition of the algorithmic resolution limit. Details can be found in the figure caption.

## 12.2 The effect of localization error on spatial point processes

We first show that a homogeneous Poisson process remains completely spatially random under the effect of localization errors. To do so, we model the resulting process as being a Neyman-Scott process (Cressie, 1993, p.664) where the parents $\{\mathbf{s}_1, \mathbf{s}_2, ...\}$ are the true positions of the events, and each parent $\mathbf{s}_i$ has

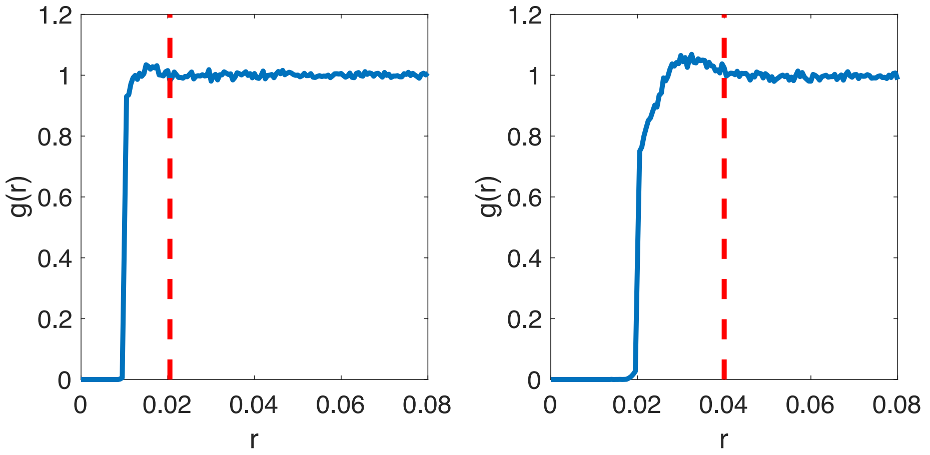


**Supplementary Figure 10.** Estimated pair correlation function (blue) and radius of correlation (red) for HPP $N_{\mathrm{O}}$ subject to imaging operator as described in Model 3 for (left) $\alpha = 0.01$ and (right) $\alpha = 0.02$. For each, 2000 independent realizations of an HPP with intensity $\lambda = 500$ per unit area were simulated. Pair correlation functions on each simulation were estimated (see *Methods* for details) and averaged together to give the plotted pair correlation estimate. The radius of correlation was estimated using the method outlined in Section 9, giving $\hat{\alpha} = \hat{\rho}/2 = 0.0105$ and $0.0200$, respectively.

exactly one offspring $\mathbf{u}_i$ – the localization – that is placed a random vector $\boldsymbol{\epsilon}_i$ away such that $\mathbf{u}_i = \mathbf{s}_i + \boldsymbol{\epsilon}_i$. The observed events are $\{\mathbf{u}_1, \mathbf{u}_2, ...\}$. Using the result (8.5.39) in (Cressie, 1993, p. 665), it follows that the Ripley's $K$-function has the form $K(r) = \pi r^2$, implying complete spatial randomness.

To demonstrate that clustered processes remain clustered we use the example of the Thomas cluster process – see Section 6 – that are a standard model used in microscopy (Rubin-Delanchy et al., 2015; Griffié et al., 2017). Suppose clusters have covariance $\sigma_c^2 I_2$, and each event is subject to independent measurement errors of covariance $\sigma_\epsilon^2 I_2$, then the process remains Thomas with each cluster now having covariance $(\sigma_c^2 + \sigma_\epsilon^2) I_2$.

## 12.3 Proof of Proposition 1

**Proposition 1.** *Let $N_{\mathrm{O}}$ be a HPP and let $I_\alpha$ be the stationary and isotropic imaging operator stated in Model 2. Resulting image process $N_{I_\alpha}$ exhibits correlation over a distance of $2\alpha$, i.e. $N_{I_\alpha}(\mathrm{d}\mathbf{s})$ is uncorrelated with $N_{I_\alpha}(\mathrm{d}\mathbf{u})$ if and only if $\|\mathbf{s} - \mathbf{u}\| \geq 2\alpha$.*

*Proof.* Under this stationary and isotropic imaging operator, we assume there is a distance $\alpha$ such that

$$\mathbb{P}(N_I(\mathrm{d}\mathbf{s}) = 1 | N_{\mathrm{O}}(\mathrm{d}\mathbf{s}) = 1, N_{\mathrm{O}}(b(\mathbf{s}, \alpha)) = 1) = 1 \tag{61}$$

$$\mathbb{P}(N_I(\mathrm{d}\mathbf{s}) = 1 | N_{\mathrm{O}}(\mathrm{d}\mathbf{s}) = 1, N_{\mathrm{O}}(b(\mathbf{s}, \alpha)) > 1) = p < 1. \tag{62}$$

We wish to show that $N_I(\mathrm{d}\mathbf{s})$ and $N_I(\mathrm{d}\mathbf{u})$ are correlated for $\|\mathbf{s} - \mathbf{u}\| \leq 2\alpha$ and uncorrelated for $\|\mathbf{s} - \mathbf{u}\| > 2\alpha$. To do so, it suffices to show $E\{N_I(\mathrm{d}\mathbf{s}) N_I(\mathrm{d}\mathbf{u})\} = E\{N_I(\mathrm{d}(\mathbf{s})\} E\{N_I(\mathrm{d}\mathbf{u})\}$ if an only if $\|\mathbf{s} - \mathbf{u}\| > 2\alpha$. It

will become necessary to consider the first-order intensity of $N_I$, which is given as

$$\lambda_I \nu(\mathrm{d}\mathbf{s}) = \mathbb{P}(N_I(\mathrm{d}\mathbf{s}) = 1) \tag{63}$$

$$= \mathbb{P}(N_I(\mathrm{d}\mathbf{s}) = 1 | N_{\mathrm{O}}(\mathrm{d}\mathbf{s}) = 1)\mathbb{P}(N_{\mathrm{O}}(\mathrm{d}\mathbf{s}) = 1) \tag{64}$$

$$= (p(1-q) + q)\lambda_{\mathrm{O}}\nu(\mathrm{d}\mathbf{s}) \tag{65}$$

where $q = \exp(-\lambda_{\mathrm{O}}\pi\alpha^2)$ is the probability that given $N_{\mathrm{O}}(\mathrm{d}\mathbf{s}) = 1$ there is no further event of $N_{\mathrm{O}}$ within the ball $b(\mathbf{s}, \alpha)$. It follows that

$$E\{N_I(\mathrm{d}\mathbf{s})\}E\{N_I(\mathrm{d}\mathbf{u})\} = (p(1-q) + q)^2\lambda_{\mathrm{O}}^2\nu(\mathrm{d}\mathbf{s})\nu(\mathrm{d}\mathbf{u}). \tag{66}$$

Let us consider $E\{N_I(\mathrm{d}\mathbf{s})N_I(\mathrm{d}\mathbf{u})\}$.

$$\begin{aligned}
E\{N_I(\mathrm{d}\mathbf{s})N_I(\mathrm{d}\mathbf{u})\} &= \mathbb{P}(N_I(\mathrm{d}\mathbf{s}) = 1, N_I(\mathrm{d}\mathbf{u}) = 1) \\
&= \mathbb{P}(N_I(\mathrm{d}\mathbf{s}) = 1, N_I(\mathrm{d}\mathbf{u}) = 1 | N_{\mathrm{O}}(\mathrm{d}\mathbf{s}) = 1, N_{\mathrm{O}}(\mathrm{d}\mathbf{u}) = 1)\lambda_{\mathrm{O}}^2\nu(\mathrm{d}\mathbf{s})\nu(\mathrm{d}\mathbf{u}) \\
&= \lambda_{\mathrm{O}}^2\mathbb{P}(N_I(\mathrm{d}\mathbf{s}) = 1, N_I(\mathrm{d}\mathbf{u}) = 1 | N_{\mathrm{O}}(\mathrm{d}\mathbf{s}) = 1, N_{\mathrm{O}}(\mathrm{d}\mathbf{u}) = 1, N_{\mathrm{O}}(b(\mathbf{s},\alpha)) = 1, N_{\mathrm{O}}(b(\mathbf{u},\alpha)) = 1) \\
&\qquad \times \mathbb{P}(N_{\mathrm{O}}(b(\mathbf{s},\alpha)) = 1, N_{\mathrm{O}}(b(\mathbf{u},\alpha)) = 1)\nu(\mathrm{d}\mathbf{s})\nu(\mathrm{d}\mathbf{u}) \\
&+ \lambda_{\mathrm{O}}^2\mathbb{P}(N_I(\mathrm{d}\mathbf{s}) = 1, N_I(\mathrm{d}\mathbf{u}) = 1 | N_{\mathrm{O}}(\mathrm{d}\mathbf{s}) = 1, N_{\mathrm{O}}(\mathrm{d}\mathbf{u}) = 1, N_{\mathrm{O}}(b(\mathbf{s},\alpha)) > 1, N_{\mathrm{O}}(b(\mathbf{u},\alpha)) = 1) \\
&\qquad \times \mathbb{P}(N_{\mathrm{O}}(b(\mathbf{s},\alpha)) > 1, N_{\mathrm{O}}(b(\mathbf{u},\alpha)) = 1)\nu(\mathrm{d}\mathbf{s})\nu(\mathrm{d}\mathbf{u}) \\
&+ \lambda_{\mathrm{O}}^2\mathbb{P}(N_I(\mathrm{d}\mathbf{s}) = 1, N_I(\mathrm{d}\mathbf{u}) = 1 | N_{\mathrm{O}}(\mathrm{d}\mathbf{s}) = 1, N_{\mathrm{O}}(\mathrm{d}\mathbf{u}) = 1, N_{\mathrm{O}}(b(\mathbf{s},\alpha)) = 1, N_{\mathrm{O}}(b(\mathbf{u},\alpha)) > 1) \\
&\qquad \times \mathbb{P}(N_{\mathrm{O}}(b(\mathbf{s},\alpha)) = 1, N_{\mathrm{O}}(b(\mathbf{u},\alpha)) > 1)\nu(\mathrm{d}\mathbf{s})\nu(\mathrm{d}\mathbf{u}) \\
&+ \lambda_{\mathrm{O}}^2\mathbb{P}(N_I(\mathrm{d}\mathbf{s}) = 1, N_I(\mathrm{d}\mathbf{u}) = 1 | N_{\mathrm{O}}(\mathrm{d}\mathbf{s}) = 1, N_{\mathrm{O}}(\mathrm{d}\mathbf{u}) = 1, N_{\mathrm{O}}(b(\mathbf{s},\alpha)) > 1, N_{\mathrm{O}}(b(\mathbf{u},\alpha)) > 1) \\
&\qquad \times \mathbb{P}(N_{\mathrm{O}}(b(\mathbf{s},\alpha)) > 1, N_{\mathrm{O}}(b(\mathbf{u},\alpha)) > 1)\nu(\mathrm{d}\mathbf{s})\nu(\mathrm{d}\mathbf{u})
\end{aligned} \tag{67}$$

Through symmetry, $\mathbb{P}(N_{\mathrm{O}}(b(\mathbf{s},\alpha)) > 1, N_{\mathrm{O}}(b(\mathbf{u},\alpha)) = 1) = \mathbb{P}(N_{\mathrm{O}}(b(\mathbf{s},\alpha)) = 1, N_{\mathrm{O}}(b(\mathbf{u},\alpha)) > 1)$ and we can reduce the above to

$$\begin{aligned}
E\{N_I(\mathrm{d}\mathbf{s})N_I(\mathrm{d}\mathbf{u})\} = \lambda_{\mathrm{O}}^2(&\mathbb{P}(N_{\mathrm{O}}(b(\mathbf{s},\alpha)) = 1, N_{\mathrm{O}}(b(\mathbf{u},\alpha)) = 1) + 2p\mathbb{P}(N_{\mathrm{O}}(b(\mathbf{s},\alpha)) = 1, N_{\mathrm{O}}(b(\mathbf{u},\alpha)) > 1) \\
&+ p^2\mathbb{P}(N_{\mathrm{O}}(b(\mathbf{s},\alpha)) > 1, N_{\mathrm{O}}(b(\mathbf{u},\alpha)) > 1))\nu(\mathrm{d}\mathbf{s})\nu(\mathrm{d}\mathbf{u}).
\end{aligned} \tag{68}$$

Let us now consider the case when $\|\mathbf{s} - \mathbf{u}\| > 2\alpha$. In this circumstance, $b(\mathbf{s},\alpha) \cap b(\mathbf{u},\alpha) = \varnothing$, which due to $N_{\mathrm{O}}$ being an HPP implies $N_{\mathrm{O}}(b(\mathbf{u},\alpha))$ are $N(b(\mathbf{u},\alpha))$ independent, meaning $\mathbb{P}(N_{\mathrm{O}}(b(\mathbf{s},\alpha)) =$

$n, N_{\mathrm{O}}(b(\mathbf{u},\alpha)) = m) = \mathbb{P}(N_{\mathrm{O}}(b(\mathbf{s},\alpha)) = n)\mathbb{P}(N_{\mathrm{O}}(b(\mathbf{u},\alpha)) = m)$, $n, m \geq 0$. This in turn implies

$$E\{N_I(\mathrm{d}\mathbf{s})N_I(\mathrm{d}\mathbf{u})\} = \lambda_{\mathrm{O}}^2(q^2 + 2pq(1-q) + p^2(1-q)^2)\nu(\mathrm{d}\mathbf{s})\nu(\mathrm{d}\mathbf{u}) \tag{69}$$

$$= \lambda_{\mathrm{O}}^2(p(1-q)+q)^2\nu(\mathrm{d}\mathbf{s})\nu(\mathrm{d}\mathbf{u}). \tag{70}$$

From Supplementary Equation (66) we conclude that $\|\mathbf{s}-\mathbf{u}\| > 2\alpha$ implies $E\{N_I(\mathrm{d}\mathbf{s})N_I(\mathrm{d}\mathbf{u})\} = E\{N_I(\mathrm{d}\mathbf{s})\}E\{N_I(\mathrm{d}\mathbf{u})\}$. To complete the argument we consider the case when $\|\mathbf{s}-\mathbf{u}\| \leq 2\alpha$. In this circumstance, $b(\mathbf{s},\alpha) \cap b(\mathbf{u},\alpha) \neq \varnothing$ which means $N_{\mathrm{O}}(b(\mathbf{u},\alpha))$ is dependent on $N_{\mathrm{O}}(b(\mathbf{u},\alpha))$ and $\mathbb{P}(N_{\mathrm{O}}(b(\mathbf{s},\alpha)) = n, N_{\mathrm{O}}(b(\mathbf{u},\alpha)) = m) \neq \mathbb{P}(N_{\mathrm{O}}(b(\mathbf{s},\alpha)) = n)\mathbb{P}(N_{\mathrm{O}}(b(\mathbf{u},\alpha)) = m)$. Due to $N_{\mathrm{O}}$ being an HPP, we have

$$\mathbb{P}(N_{\mathrm{O}}(b(\mathbf{s},\alpha)) = 1, N_{\mathrm{O}}(b(\mathbf{u},\alpha)) = 1) = q^2 r \tag{71}$$

$$\mathbb{P}(N_{\mathrm{O}}(b(\mathbf{s},\alpha)) = 1, N_{\mathrm{O}}(b(\mathbf{u},\alpha)) > 1) = qr(1-q) \tag{72}$$

$$\mathbb{P}(N_{\mathrm{O}}(b(\mathbf{s},\alpha)) > 1, N_{\mathrm{O}}(b(\mathbf{u},\alpha)) > 1) = 1 - qr(2-q) \tag{73}$$

where $r = \exp(\lambda_{\mathrm{O}} R_\alpha(\|\mathbf{s}-\mathbf{u}\|))$, $R_\alpha(\|\mathbf{s}-\mathbf{u}\|)$ being the area of intersection of two circles of radius $\alpha$ a distance $\|\mathbf{s}-\mathbf{u}\|$ apart. Supplementary Equation (68) therefore becomes

$$E\{N_I(\mathrm{d}\mathbf{s})N_I(\mathrm{d}\mathbf{u})\} = \lambda_{\mathrm{O}}^2(q^2 r + 2pqr(1-q) + p^2(1 - qr(2-q)))\nu(\mathrm{d}\mathbf{s})\nu(\mathrm{d}\mathbf{u}) \tag{74}$$

To show (66) and (68) are not equal we subtract one from the other, giving

$$E\{N_I(\mathrm{d}\mathbf{s})N_I(\mathrm{d}\mathbf{u})\} - E\{N_I(\mathrm{d}\mathbf{s})\}E\{N_I(\mathrm{d}\mathbf{u})\} = \lambda_{\mathrm{O}}^2(1-r)((p(1-q)+q)^2 - p^2). \tag{75}$$

The right-hand side equals zero only when either (i) $r = 1$, or (ii) $p = p(1-q)+q \implies pq = q \implies p = 1$ or $q = 0$. Condition (i) is only satisfied when $R_\alpha(\|\mathbf{s}-\mathbf{u}\|) = 0 \implies \|\mathbf{s}-\mathbf{u}\| \geq 2\alpha$. Condition (ii) can not be satisfied under the assumptions of the model, completing the proof. □

## 12.4 Proof for Proposition 4

**Proposition 4.** Let $N_{\mathrm{O}}$ be a spatial point process. Consider a single molecule localisation microscopy experiment with assumptions 1-3 as outlined above. As the number of frames $n_{\mathrm{F}} \to \infty$ and $q \to 0$ such that $qn_{\mathrm{F}} = 1$, for $\mathbf{s} \in \Phi_{\mathrm{O}}$, $\mathbb{P}(\mathbf{s} \in \Phi_{\mathrm{R}}) \to 1$.

*Proof.* For a given $\mathbf{s} \in \Phi_{\mathrm{O}}$,

$$
\begin{aligned}
\mathbb{P}(\mathbf{s} \in \Phi_R) &= \sum_{i=1}^{n_{\mathrm{F}}} \mathbb{P}(\mathbf{s} \in \Phi_R^i | \mathbf{s} \in \Phi^i)\mathbb{P}(\mathbf{s} \in \Phi^i) \\
&= qn_{\mathrm{F}}\mathbb{P}(\mathbf{s} \in \Phi_R^i | \mathbf{s} \in \Phi^i) \\
&= \mathbb{P}(\mathbf{s} \in \Phi_R^i | \mathbf{s} \in \Phi^i) \qquad\qquad (qn_{\mathrm{F}} = 1) \\
&= \mathbb{P}(N_q(b(\mathbf{s}, \alpha)) = 1) \\
&= \sum_{n=1}^{\infty} \mathbb{P}(N_q(b(\mathbf{s}, \alpha)) = 1 | N_{\mathrm{O}}(b(\mathbf{s}, \alpha)) = n)\mathbb{P}(N_{\mathrm{O}}(b(\mathbf{s}, \alpha)) = n) \\
&= \mathbb{P}(N_{\mathrm{O}}(b(\mathbf{s}, \alpha)) = 1) + \sum_{n=2}^{\infty}(1-q)^{n-1}\mathbb{P}(N_{\mathrm{O}}(b(\mathbf{s}, \alpha)) = n) \\
&= \mathbb{P}(N_{\mathrm{O}}(b(\mathbf{s}, \alpha)) = 1) + \sum_{n=2}^{\infty}\sum_{k=0}^{n-1}(-1)^k \begin{pmatrix} n-1 \\ k \end{pmatrix} q^k \mathbb{P}(N_{\mathrm{O}}(b(\mathbf{s}, \alpha)) = n) \\
&= \sum_{n=1}^{\infty} \mathbb{P}(N_{\mathrm{O}}(b(\mathbf{s}, \alpha)) = n) + \sum_{n=2}^{\infty}\sum_{k=1}^{n-1}(-1)^k \begin{pmatrix} n-1 \\ k \end{pmatrix} q^k \ \mathbb{P}(N_{\mathrm{O}}(b(\mathbf{s}, \alpha)) = n) \\
&= 1 + \sum_{n=2}^{\infty}\sum_{k=1}^{n-1}(-1)^k \begin{pmatrix} n-1 \\ k \end{pmatrix} q^k \ \mathbb{P}(N_{\mathrm{O}}(b(\mathbf{s}, \alpha)) = n) \longrightarrow 1, \quad \text{as } q \to 0. \qquad (76)
\end{aligned}
$$

□

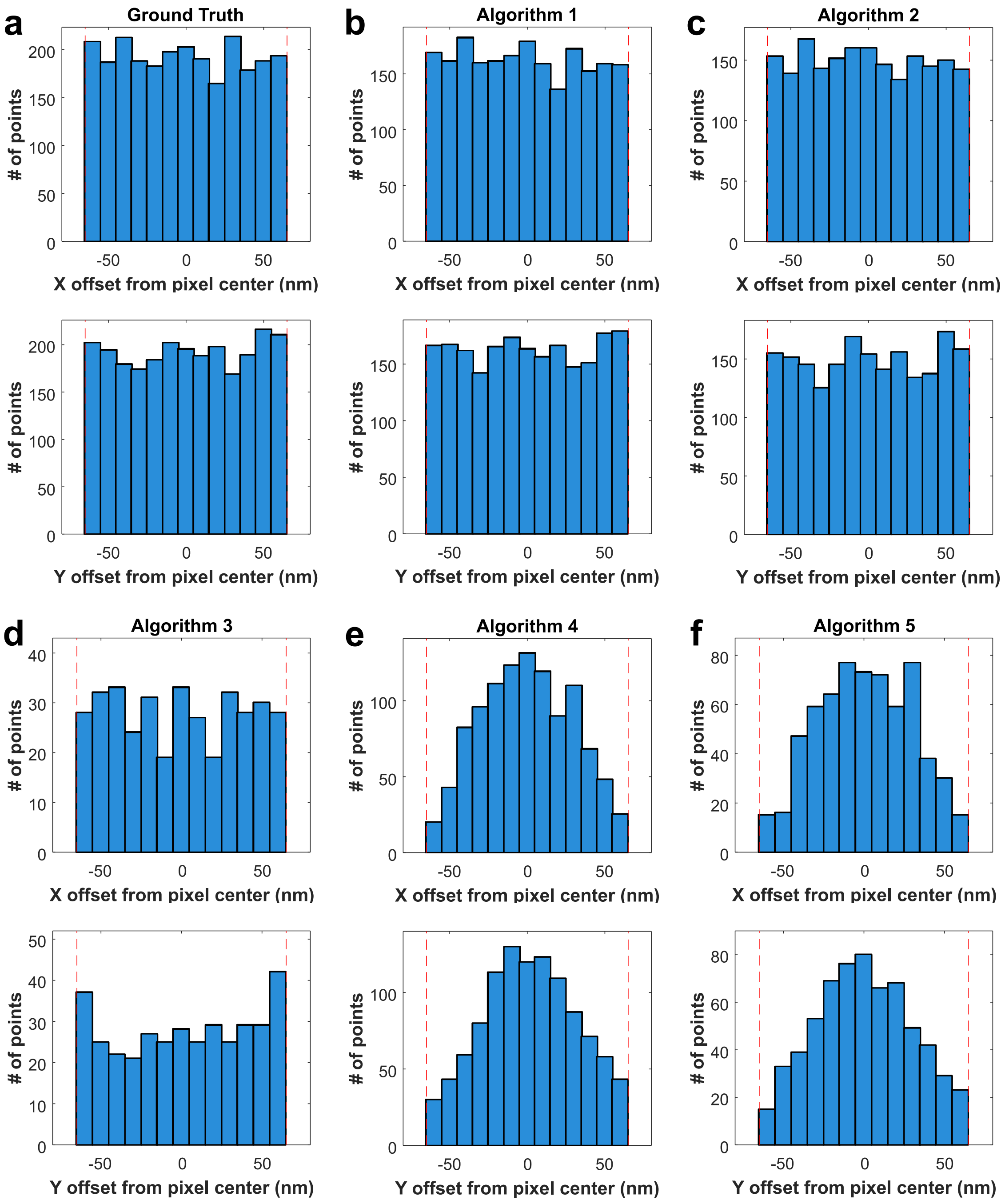


**Supplementary Figure 11.** Distribution of the ground-truth locations of detected molecules relative to the center of pixels varies between image analysis algorithms. For each of the indicated image analysis algorithms, the offset of the ground-truth location coordinate of each molecule detected by an algorithm from the center of the detector pixel the molecule is located within was calculated. The distributions of these offsets for each image analysis algorithm across the width and height of a pixel are plotted here for the image shown in Figure 2a and analyzed in Figure 2b. Since the object locations in Figure 2a are simulated with a completely spatially random distribution, the distribution of these offsets is expected to be uniform, as shown **a**. However, the Algorithm 4 (**e**) and Algorithm 5 (**f**) detect more molecules that are closer to the center of pixels compared to those located closer to the edge of pixels unlike the other image analysis algorithms we examined.

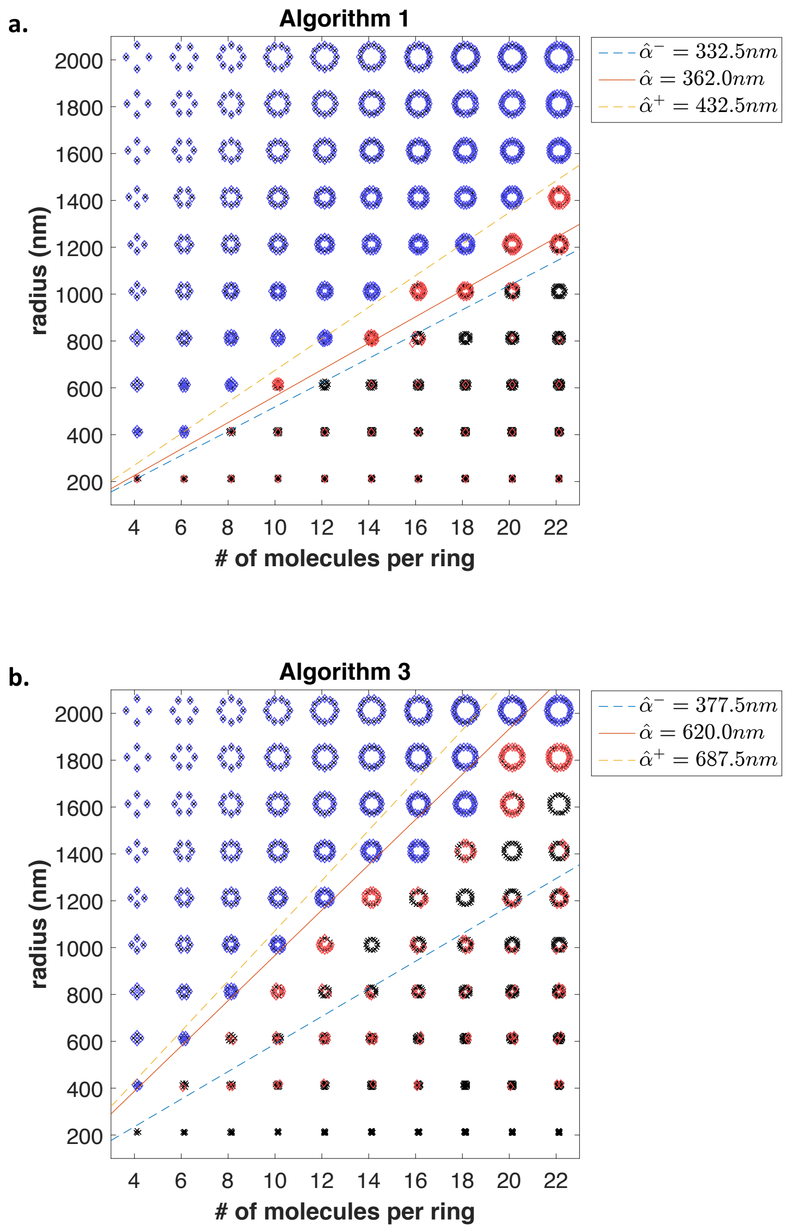


**Supplementary Figure 12.** The resolution limit of the indicated analysis approach is illustrated here using images of deterministic structures similar to Figure 3. Each structure consists of single molecules positioned evenly around the edge of a ring, indicated by crosses. The x and y axes indicate the number of molecules comprising each ring structure and its radius respectively. Localizations obtained by the analysis approach, indicated by diamonds, corresponding to structures where all constituent molecules were accurately identified and localized to within 10nm of the true position are shown in blue. Localizations corresponding to structures where one or more molecules were either not identified or where the localization deviated by more than 10nm from the true location are shown in red. The solid line indicates the estimated algorithmic resolution limit $\hat{\alpha}$. The dashed lines show the 80% bootstrapped confidence interval for the estimated $\hat{\alpha}$. All structures with single molecules separated by a distance larger than the algorithmic resolution limit, i.e., to the left of the lines corresponding to $\hat{\alpha}$, are accurately recovered.

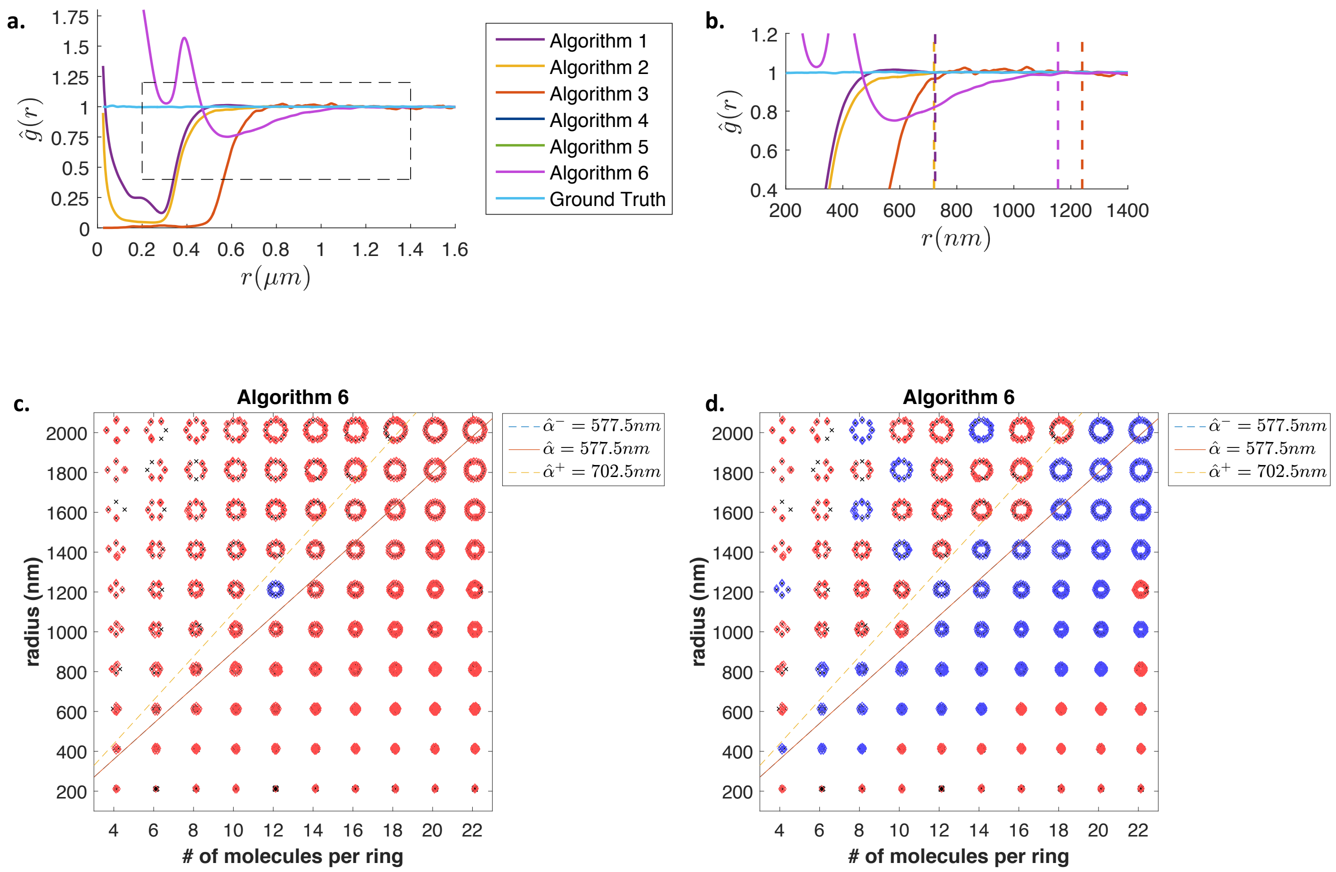


**Supplementary Figure 13.** Results from applying Algorithm 6 which employs a multi-emitter analysis approach. **(a)** The results shown in Figure 2d augmented with the pair-correlation plot corresponding to Algorithm 6. The pair-correlations results for Algorithm 6 were calculated based on the localizations obtained by analyzing the same images used in Figure 2d. **(b)** Magnified view of the region in **a** indicated by the dashed rectangle. The dashed, vertical lines indicate the algorithmic resolution limits. **(c)** and **(d)** The results of using Algorithm 6 to analyze the same images of deterministic structures analyzed in Figure 3. Each structure consists of single molecules positioned evenly around the edge of a ring, indicated by crosses. The x and y axes indicate the number of molecules comprising each ring structure and its radius respectively. Localizations obtained by the analysis approach are indicated by diamonds. The solid line indicates the algorithmic resolution limit $\hat{\alpha}$ estimated from the pair-correlation results shown in **a**. The dashed lines show the 80% bootstrapped confidence interval for the estimated $\hat{\alpha}$ in **b**. Localizations obtained using Algorithm 6 corresponding to structures where all constituent molecules were accurately identified and localized to within 10nm of the true position without any repeat detection of a particular molecule are shown in blue. Localizations corresponding to structures where one or more molecules were either not identified, where the localization deviated by more than 10nm from the true location, or where the number of molecules in the structure was overestimated by the algorithm are shown in red. In **c**, the localizations obtained in **b** are plotted again such that repeat detections of a particular molecule are ignored when determining whether a structure was recovered by the algorithm or not. Therefore, localizations corresponding to structures were all constituent molecules are identified at least once and localized to within 10nm of the true position are shown in blue while localizations corresponding to all other structures are shown in red.

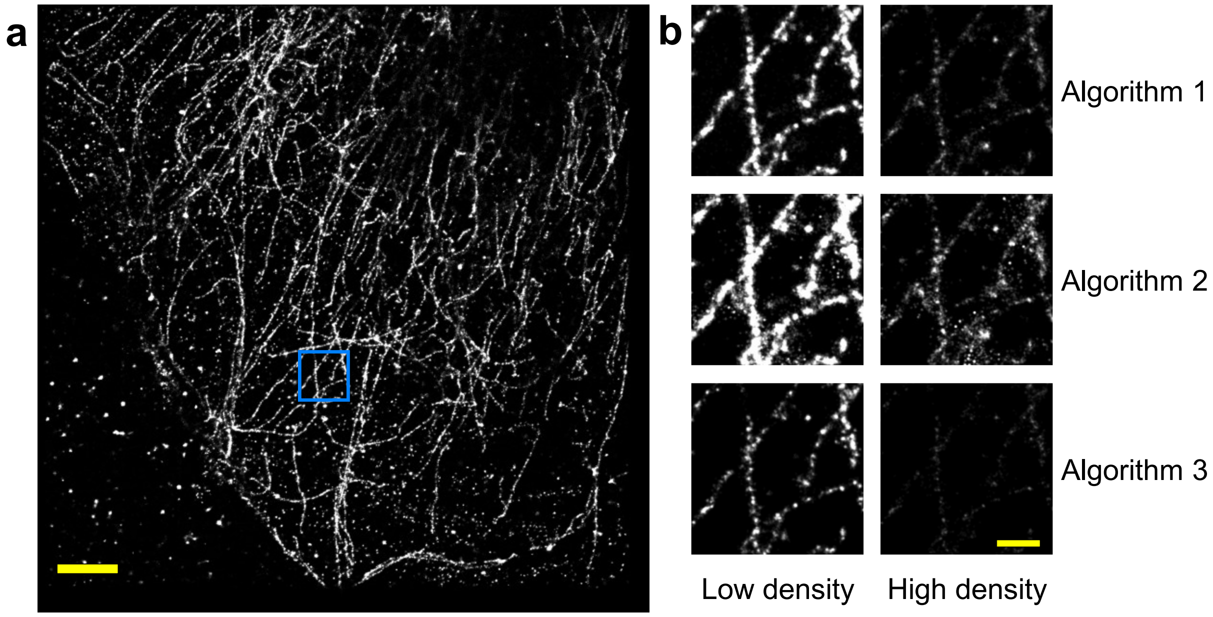


**Supplementary Figure 14.** Results from analyzing both low and high-density super-resolution images of tubulin acquired from a BS-C-1 cell. (**a**) A super-resolution reconstruction of the distribution of tubulin in a BS-C-1 cell. The image is reconstructed using the localizations obtained by analyzing low-density super-resolution images using Algorithm 1. The reconstruction was generated by simulating Gaussian profiles centered at each valid localization produced by Algorithm 1 using $\sigma = 17$nm for the width of the Gaussian PSF. The value for $\sigma$ was selected by calculating the limit of the localization accuracy (Ober et al., 2004; Ram et al., 2006) for the corresponding experimental and imaging parameters, using the average number of photons detected from each fluorophore localized by Algorithm 1, and the average noise associated with the signal from each fluorophore. The blue box indicates the region displayed in higher magnification in the adjacent panel. Scalebar (yellow) = 5μm. (**b**) Magnified view of super-resolution reconstructions of the region marked in a using the localizations produced by the indicated algorithms. For all three algorithms, more localizations are obtained when analyzing the low-density images compared to the high-density images resulting in better clarity in the reconstructions corresponding to the low-density images. Scalebar (yellow) = 1μm.

## Supplementary References

M. Afshang, C. Saha, and H. Dhillon. Nearest-neighbor and contact distance distributions for thomas cluster process. *IEEE Wireless Communication Letters*, 6:130 – 133, 2016.

A. Al-Hourani, R. J. Evans, and S. Kandeepan. Nearest-neighbor distance distribution in hard-core point processes. *IEEE Communication Letters*, 20:1872 — 1875, 2016.

R. W. R. Baker and J. A. Nissim. Expressions for combining standard errors of two groups and for sequential standard error. *Nature*, 198:1020, 1963.

N. A. C. Cressie. *Statistics for spatial data*. John Wiley and Sons, Inc., 1993.

P. J. Diggle. *Statistical Analysis of Spatial Point Patterns*. Academic Press, 1983.

B. Efron and R.J. Tibshirani. *An introduction to the Bootstrap*. Chapman & Hall, New York, 1993.

J. Griffié, L. Shlomovich, D. Williamson, J. Aarons M. Shannon, S. Khuon, G. Burn, L. Boelen, R. Peters, A. Cope, E. A. K. Cohen, P. Rubin-Delanchy, and D. M. Owen. 3D Bayesian cluster analysis of super-resolution data reveals lat recruitment to the t cell synapse. *Scientific Reports*, 7:4077, 2017.

J. Illian, A. Penttinen, H. Stoyan, and D. Stoyan. *Statistical Analysis and Modelling of Spatial Point Patterns*. John Wiley & Sons, Ltd, 2008.

T. Lagache, G. Lang, N. Sauvonnet, and JC. Olivo-Marin. Analysis of the spatial organization of molecules with robust statistics. *PLOS one*, 8(12):e80914, 2013.

B. Matérn. Spatial variation. *Lecture Notes in Statistics*, 36, 1960.

R. J. Ober, S. Ram, and E. S. Ward. Localization accuracy in single-molecule microscopy. *Biophysical Journal*, 86:1185 — 1200, 2004.

M. Penrose. *Random Geometric Graphs*. Oxford University Press, 2003.

S. Ram, E. S. Ward, and R. J. Ober. Beyond rayleigh's criterion: A resolution measure with application to single-molecule microscopy. *Proceedings of the National Academy of Science*, 103:4457 — 4462, 2006.

B. D. Ripley. *Spatial Statistics*. Wiley, New York, 1981.

J. Rossy, E. A. K. Cohen, K. Gaus, and D. M. Owen. Method for co-cluster analysis in multichannel single molecule localization data. *Histochemisty and Cell Biology*, 141:605 — 612, 2014.

P. Rubin-Delanchy, G. L. Burn, J. Griffié, D. J. Williamson, N. A. Heard, and D. M. Owen A. P. Cope. Bayesian cluster identification in single-molecule localization microscopy data. *Nature Methods*, 12:1072 — 1076, 2015.

D. Sage, H. Kirshner, T. Pengo, N. Stuurman, J. Min, S. Manley, and M. Unser. Quantitative evaluation of software packages for single-molecule localization microscopy. *Nature Methods*, 12:717 — 724, 2015.

D. Stoyan and H. Stoyan. On one of Matérn's hard-core point process models. *Math. Nachr.*, 122:205 —

214, 1985.

D. Stoyan, W. S. Kendall, and J. Mecke. *Stochastic Geometry and its Applications*. John Wiley & Sons, Ltd. 2nd Edition, 1995.
